# A machine learning approach to identify important variables for distinguishing between fallers and non-fallers in older women

**DOI:** 10.1371/journal.pone.0293729

**Published:** 2023-10-31

**Authors:** Emily Gregg, Clive Beggs, Athanassios Bissas, Gareth Nicholson

**Affiliations:** 1 Carnegie School of Sport, Leeds Beckett University, Leeds, United Kingdom; 2 York Health Economics Consortium, University of York, York, United Kingdom; 3 Department of Medicine for the Elderly, Cambridge University Hospitals, Cambridge, United Kingdom; 4 School of Sport and Exercise, University of Gloucestershire, Gloucester, United Kingdom; University of Innsbruck, AUSTRIA

## Abstract

Falls are a significant ongoing public health concern for older adults. At present, few studies have concurrently explored the influence of multiple measures when seeking to determine which variables are most predictive of fall risks. As such, this cross-sectional study aimed to identify those functional variables (i.e. balance, gait and clinical measures) and physical characteristics (i.e. strength and body composition) that could best distinguish between older female fallers and non-fallers, using a machine learning approach. Overall, 60 community-dwelling older women (≥65 years), retrospectively classified as fallers (n = 21) or non-fallers (n = 39), attended three data collection sessions. Data (281 variables) collected from tests in five separate domains (balance, gait, clinical measures, strength and body composition) were analysed using random forest (RF) and leave-one-variable-out partial least squares correlation analysis (LOVO PLSCA) to assess variable importance. The strongest discriminators from each domain were then aggregated into a multi-domain dataset, and RF, LOVO PLSCA, and logistic regression models were constructed to identify the important variables in distinguishing between fallers and non-fallers. These models were used to classify participants as either fallers or non-fallers, with their performance evaluated using receiver operating characteristic (ROC) analysis. The study found that it is possible to classify fallers and non-fallers with a high degree of accuracy (e.g. logistic regression: sensitivity = 90%; specificity = 87%; AUC = 0.92; leave-one-out cross-validation accuracy = 63%) using a combination of 18 variables from four domains, with the gait and strength domains being particularly informative for screening programmes aimed at assessing falls risk.

## Introduction

Falls are not an inevitable part of ageing, but they are a significant public health concern for older adults. Whilst falls have been the focus of extensive work to date, they still pose a serious clinical problem worldwide [1]. It is estimated that one in three adults aged >65 years fall each year, and older women have an increased risk of falls compared with older men [2].

Identifying risk factors that predispose individuals to fall is an important aspect of falls prevention and management [3]. Over past decades, a significant body of research has been dedicated to investigating falls, and more than 400 risk factors have been proposed [4]. Previous research has confirmed that falls are complex and multifactorial in nature, with an extensive range of intrinsic (e.g. balance, gait, strength) and extrinsic (e.g. environmental, footwear) features identified [5, 6].

To enable the accurate identification of high-risk individuals, the most important modifiable risk factors and their ability to predict falls need to be determined. This information can help inform the design of effective screening tools alongside prevention and rehabilitation interventions. Impaired balance, gait and mobility, as well as the underpinning age-related changes in body composition and muscle function, have been presented as key domains related to falls [5, 7]. Various studies have investigated the ability of variables within these domains to differentiate between fallers and non-fallers; however, there remain many conflicting results. For example, some authors reported that several balance and gait variables can differentiate between fallers and non-fallers [8–10], whereas others observed no differences between groups for similar variables [11, 12]. In terms of muscle strength, inconsistent findings across different contraction types and muscle groups have been observed with isometric [12] and isokinetic [13] protocols displaying differences in their ability to discriminate between fallers and non-fallers. The contrasting findings of previous investigations creates uncertainty regarding the discriminatory ability of different variables. This presents serious challenges for optimal screening and targeted falls prevention interventions in this population.

Although differences in study design, population characteristics, and testing protocols have likely contributed to the inconsistencies outlined, much of the previous work has only focused on variables from one single domain (i.e. gait, balance or strength) [9, 13]. This approach neglects the multifactorial nature of falls. Where studies have considered more than one domain (e.g. balance, gait, strength) [14, 15], only a limited number of variables from each domain have been included, and there is a lack of information regarding mechanical and neuromuscular factors which underpin the measured variables. Additionally, there is limited consideration of the relative importance of the variables regarding their predictive ability. Generally, traditional univariate or multivariate techniques have been used. These, however, are often unable to capture the complexity of large datasets that may exhibit considerable multicollinearity [16]. Datasets associated with falling often exhibit multicollinearity, with multiple strongly correlated variables, leading to redundancy (uninformative variables) in the data. Consequently, an excessive number of risk factors have been identified, many which overlap, which is often confusing [6].

To move beyond the often-circular nature of falls research, there is a need for a model which integrates essential variables from multiple domains [6]. Recently, innovative attempts have been made to evaluate the relative contributions of a comprehensive range of measures across strength, balance and gait domains using sophisticated analysis techniques [3, 17, 18]. These investigations are of great significance and provide preliminary evidence regarding the importance and sensitivity of gait variables in discriminating between fallers and non-fallers [17, 18]. Although these studies are more comprehensive, some important physical characteristics (e.g. body composition, rapid strength) have not been fully explored or are missing, and strength and asymmetry assessments across multiple joints/regions (i.e. ankle, knee and trunk) are lacking. Furthermore, the inclusion of a comprehensive battery of clinical measures, which are commonly used in community-based settings and clinical practice, is needed to better understand their predictive capabilities when discriminating between potential fallers and non-fallers. Consequently, to the authors’ knowledge, no comprehensive studies exist that incorporate both the important functional variables (e.g. balance and gait impairments) and a wide range of underlying physical characteristics (via the inclusion of key physical measures such as muscle strength, muscle quality (MQ), and body composition) which underpin them [6]. Including a combination of these variables is essential to understand the full picture of falls risk and the underpinning factors driving functional impairments in older adults, and women in particular.

Principal component analysis (PCA) has been used in some of these recent comprehensive studies because falls datasets often contain considerable redundancy [17, 18]. While PCA has merit, it is not possible to rank the observed variables in order of importance or eliminate redundant variables that add little value to the discrimination process. However, there have been some recent investigations using other machine learning techniques to assess feature importance in this population [3, 19–21]. For example, Qiu and colleagues [3] highlighted the benefits of applying machine learning techniques when investigating falls risk. Within their study, six machine learning techniques (random forest analysis, logistic regression, naïve bayes, decision tree, boosted tree, and support vector machine) were applied to a comprehensive range of variables (n = 155), and ten were identified as important for discriminating between fallers and non-fallers [3]. Despite the comprehensive nature of this investigation and the applications for screening and intervention design, the data were collected using wearable sensors meaning that some key domains and important variables (e.g. ground reaction force (GRF) data, muscle strength and body composition) were omitted from their analyses. As with the previous comprehensive analyses [17, 18], these missing domains/variables are essential to understand the full risk of falls in this population and to improve the effectiveness of testing batteries and falls prevention interventions.

Therefore, we undertook a comprehensive exploratory study using measured balance, gait, clinical measures, strength, and body composition variables from a cohort of older women. We used a range of sophisticated machine learning techniques to identify important and redundant variables across these domains. As such, this study sought to: a) identify the functional and physical factors that best differentiate between fallers and non-fallers in older women, and b) quantify the relative importance of these variables.

## Materials and methods

### Participants

80 community-dwelling older women (≥65 years of age) were recruited for the study: 20 of these did not meet the inclusion criteria and were excluded, leaving 60 participants who were enrolled and completed the data collection (Table 1). The sample size was deemed appropriate given the exploratory nature of the work and aligns with recent research (e.g. [17, 18]) that has conducted a comprehensive multi-domain analyses using machine learning techniques. Participants were randomly recruited from within the local community through a range of avenues, including liaising with Neighbourhood Network Schemes and the University of the Third Age. To advertise this study, several recruitment presentations were conducted, and flyers were distributed physically and online (via email and social media). Prior to the commencement of this research, ethical approval was gained from the Carnegie School of Sport Research Ethics Sub-Committee at Leeds Beckett University (approval reference 35011). Participants provided written informed consent before participating in this study. Participants were classified as ‘fallers’ or ‘non-fallers’ based on self-reported falls history. The number of fallers relative to non-fallers was consistent with the prevalence of fallers in the older adult population (~30% of older adults >65 years fall each year) [2, 22]. Participants completed health screening before taking part in this study. Several exclusion criteria were implemented to minimise any health risks to participants and to ensure safety during testing. Exclusion criteria were:

■ Any history of cardiovascular, metabolic or renal disease, or any signs and symptoms suggestive of such diseases;
■ Resting blood pressure ≥140/90 (participants prescribed blood pressure medication were asked to seek medical clearance from their GP);
■ Self-reported history of a pacemaker and/or any other internal electrical medical device;
■ Serious mobility impairment or any bone, joint or muscle problem that could have been aggravated by exercise;
■ Medical conditions that could have led to more substantial complications during maximal exercise testing;
■ Self-reported history of a serious visual impairment;
■ Excessive alcohol consumption on a regular basis defined as >14 units per week; or
■ A Mini Mental State Examination score <24.

**Table 1:** Descriptive characteristics of fallers and non-fallers included within the multi-domain analysis.

	Fallers (n = 21)	Non-fallers (n = 39)	*p* value	Effect size
Age (years)	71.52±4.33	68.87±3.41	0.02**	0.71
Mass (kg)	65.43±10.67	62.31±10.55	0.28	0.62
Height (m)	1.62±0.05	1.60±0.06	0.32	0.25
BMI (kg/m^2^)	25.13±4.06	24.25±3.6	0.41	0.23
Systolic BP (mmHg)	121.27±12.73	125.56±13.65	0.23	0.22
Diastolic BP (mmHg)	75.56±5.94	77.33±8.39	0.35	0.23
Resting HR (bpm)	69.52±7.22	69.5±6.82	0.99	0.23
Number of medications	1.14±1.06	0.92±1.11	0.46	0.20
Weekly alcohol (units)	10.6±9.61	8.06±8.13	0.31	0.29
FES-I	20.43±2.77	18.68±2.61	0.02**	0.66
GDS	1.29±1.71	1.33±2.25	0.93	0.02
Mini-Mental State Examination	29.43±0.98	29.36±0.84	0.78	0.08
Total physical activity (MET-minutes/week)	5990±3711	7463±3860	0.16	0.39
Visual impairment^△^	20 (95.2%)	36 (92.3%)	1.00	<0.001
Hearing impairment^△^	5 (23.8%)	7 (17.9%)	0.84	0.03

BMD, bone mineral density; BMI, body mass index; BP, blood pressure; HR, heart rate; FES-I, Falls Efficacy Scale—International; GDS, Geriatric Depression Scale.

Continuous variables are mean ± SD. ^△^Categorical variables are n (%). Group differences and effect sizes were determined using two-tailed t-tests and Cohen’s *d* for all continuous variables. ^△^For categorical variables, group differences and effect sizes were determined using chi-squared tests and Cramer’s V.

* *p≤*0.10

** *p≤*0.05

*** *p≤*0.001.

Fallers were classified retrospectively as having experienced one or more falls in the 12 months preceding data collection or whilst enrolled within the study. Non-fallers had no history of falls during this time. Falls were defined using the World Health Organization’s definition as “inadvertently coming to rest on the ground, floor or other lower level, excluding intentional change in position to rest in furniture, wall or other objects” [23]. Overall, 10 participants reported the occurrence of one fall, and 11 reported two or more falls.

Falls history data were recorded during a telephone conversation before the first data collection session and subsequently during each visit to the laboratory. Participants were asked to provide detailed information about each fall, including when and where the fall occurred and if any injuries were sustained. All information was verified in person during each data collection session to ensure recording accuracy.

### Experimental design

**Fig 1:**
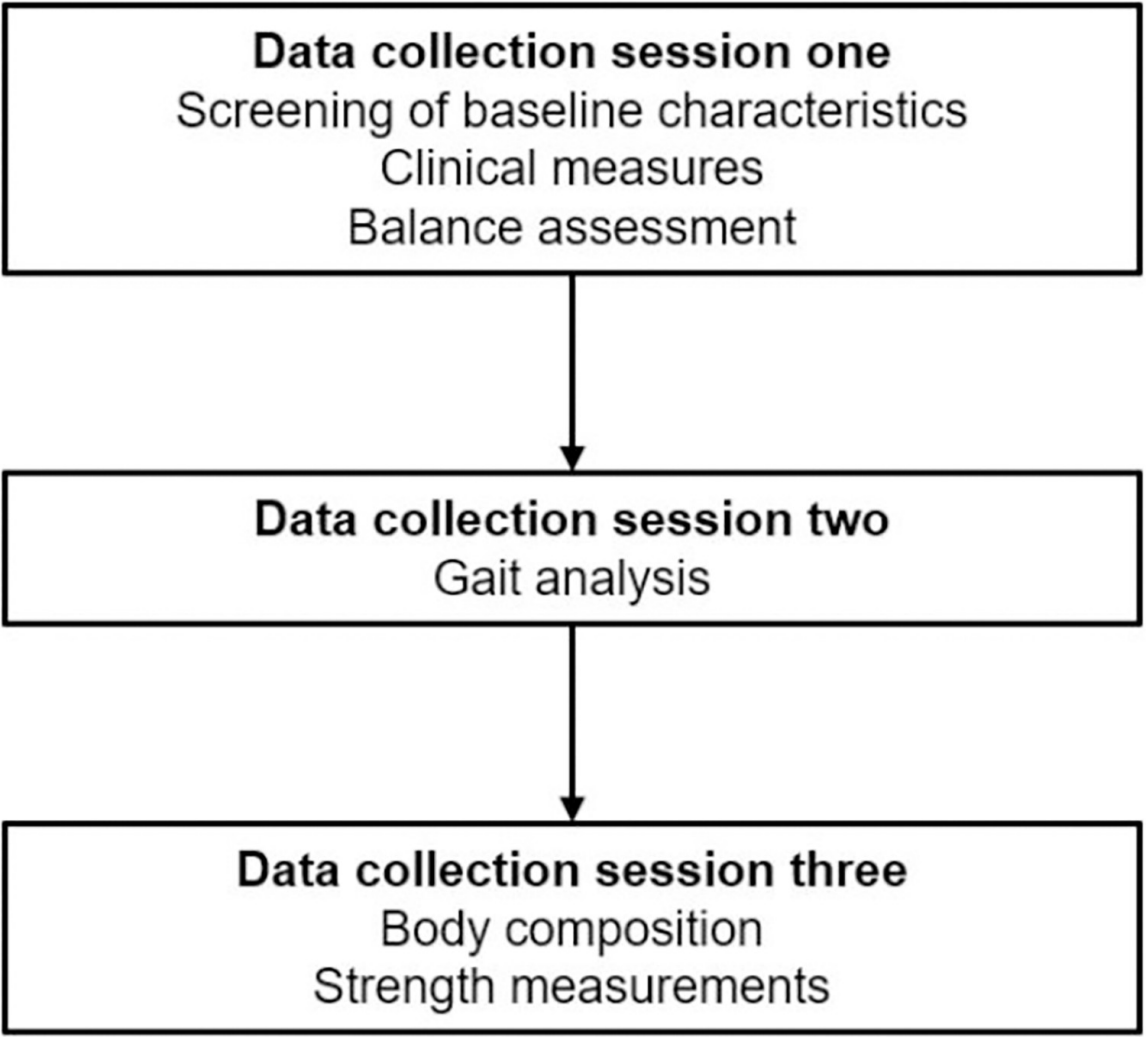
Overview of the functional and physical measures included in this project.

A cross-sectional study design was employed within this research. Participants attended three data collection sessions in the Carnegie Research Institute at Leeds Beckett University (Fig 1). Recruitment for this study opened in February 2017, and data collection took place between March 2017 and December 2018, with visits approximately two months apart. During session one, participants underwent screening of baseline characteristics, clinical measures, and balance assessment. During session two, gait analysis was performed, and during the final session, body composition and strength measurements were conducted. Before each data collection session, participants were advised to rest and to maintain a consistent nutritional intake and physical activity level. Throughout the duration of the study, participants did not engage in any new physical activities, supplementation, or falls prevention programmes (based on self-reported information at each data collection session).

### Experimental procedures

#### Clinical measures

Four clinical measures, which are commonly used in research and clinical practice with older adults, were used to evaluate functional mobility. Participants completed the following protocols in a randomised order: Timed Up and Go (TUG) [24], stair test [25], chair stand test [26], and Tinetti Performance Oriented Mobility Assessment (POMA) [27]. For each of the clinical measures, participants completed one familiarisation trial followed by two testing trials [28], with a one-minute rest period between trials [29].

#### Balance measures

Multiple static posturography protocols were performed during a single visit to the laboratory using the NeuroCom VSR SPORT force platform integrated with the Balance Manager software (NeuroCom International, Inc., Clackamas, Oregon, USA). Balance performance was quantified during five testing protocols: Modified Clinical Test of Sensory Interaction on Balance (feet apart and narrow stance) [30, 31], unilateral stance [32], limits of stability [33], and weight bearing squat [9]. Throughout all measurements, a screen was positioned at eye level, 1.5 m in front of the force plate [34]. When required, a high-density foam pad was used to create a compliant surface. Participants completed testing barefoot, facing the screen with hands on hips. A one-minute rest period was provided between trials, and a two-minute rest period was provided between protocols [35].

#### Gait measures

Kinetic and kinematic data were simultaneously collected during walking trials along a 10-meter indoor walkway at two gait speeds (self-selected usual and maximal). Participants were asked to walk at a comfortable everyday pace for the usual gait speed (UGS) trials and as quickly but safely as possible for the maximal gait speed (MGS) trials [10]. For both speeds, participants completed five familiarisation trials followed by five testing trials, with two minutes rest between trials. To determine gait speed, timing gates (Witty; Microgate, Bolzano, Italy) mounted on tripods were positioned at approximately hip height, five metres apart in the centre of the walkway. GRF data were acquired from three force platforms (Kistler Instruments Ltd., Winterthur, Switzerland) sampling at 1,000 Hz. Data were acquired using the BioWare software (version 5.3.1.7; Kistler Instruments Ltd., Winterthur, Switzerland), configured to record for 20 seconds per trial. Trials were deemed acceptable when both feet contacted separate force plates, without participants noticeably altering their gait style or targeting the plates.

Two-dimensional video data were collected from one high-speed camera (Fastec TS3; Fastec, San Diego, CA, USA) placed perpendicular to the walkway and eight metres from the centre of the force platforms. The camera settings included a frame rate of 100 Hz, shutter speed of 1/1,000 s, resolution of 1,280 x 1,024 pixels, and *f*-stop of 2.0. Before testing, tape markers were placed on the hip (lateral aspect of the greater trochanter), knee (midpoint between the lateral convexities of the femur and tibia), and ankle (lateral malleolus) joints on the right-hand side of the body to aid digitising reliability during kinematic analysis. Participants completed all trials in their own footwear. A reference frame was constructed using four metal poles placed in the sagittal plane in the centre of the walkway. This was recorded and later used for calibration.

#### Body composition measures

Dual energy X-ray absorptiometry (DXA) scans (Lunar iDXA with enCORE software version 15.0; GE Healthcare, Madison, WI, USA) were performed to assess body composition, bone mineral density (BMD), and hip structure. All scans were performed by the same trained operator. Participants were asked to arrive fasted, in a euhydrated state, and having participated in no vigorous exercise for 12 hours preceding the scans [36]. Participants were also asked to void their bladder before the scans [37]. The scanning mode was automatically determined by the enCORE software based on body size; all participants in this study were scanned using the standard mode (estimated body thickness 0.16 m to 0.25 m).

Total and regional body composition measures were derived from one total body scan, lasting approximately seven minutes [38]. Participants adopted a supine position, aligned with the centre line on the DXA scanning table, with the head positioned at the horizontal line at the top of the scanning bed. Proximal femur BMD and structural geometry were evaluated from one left femur scan [39]. For this, participants remained in a supine position in the centre of the scanning table. For any participants with history of a left hip replacement, the right femur was scanned instead (n = 2). On completion of the DXA scans, participants had the opportunity to eat and drink before moving onto the strength measurements.

#### Strength measures

Strength assessments were performed at the trunk, knee and ankle for both limbs using an isokinetic dynamometer (System 4 PRO; Biodex Corp., Shirley, New York, USA). For the knee and ankle measurements, the order of testing (joint, type of contraction and speed) was randomised. The rotational axis of the dynamometer was visually aligned with a line traversing the femoral epicondyles at the knee joint centre, and the resistance pad was positioned securely on the tibia superior to the medial malleolus [40]. The testing thigh, contralateral limb, trunk and pelvis were stabilised throughout the protocol using Velcro straps. Peak torque and rate of torque development (RTD) were measured during maximal isometric knee extension trials, with participants completing three submaximal trials before three maximal test trials. Each contraction was performed for five seconds, with a 30 second rest period between trials [41]. Maximal isokinetic concentric joint torques were assessed at the knee and ankle for both flexion and extension at angular velocities of 60°/s and 120°/s [42, 43]. Participants completed five submaximal trials followed by three maximal test trials. Following these measurements, concentric trunk flexion and extension data under isokinetic conditions were collected at angular velocities of 20°/s and 45°/s [44] using the Biodex Dual Position Back Extension/Flexion attachment (Biodex Corp., Shirley, New York, USA) attached to the dynamometer. The fixed axis of the dynamometer was aligned level with the anterior superior iliac spines [45]. Participants performed five submaximal warm up trials before five maximal test trials. For the isokinetic trials, a one-minute rest period was provided between sets.

### Data analysis

#### Clinical measures data

For each of the clinical measures (except gait speed), the average performance across both test trials was calculated and used within the analysis [46]. In total, ten variables were obtained from the clinical data protocols (Table 2). Gait speed data were collected alongside the other gait variables but included within the clinical measures dataset, given their ease of measurement and common use in clinical and community settings. Further details are provided in the gait data section below.

**Table 2:** Variables included within the clinical measures, balance, gait, strength and body composition data packages for the single-domain analyses.

**Clinical measures variables**	
TUG	• Time (s)
Stair test	• Stair ascent time (s) • Stair descent time (s)
Chair stand test	• Performance (count)
Gait speed	• UGS (m/s) • MGS (m/s) • Gait speed reserve
Tinetti POMA	• Balance score • Gait score • Total score
**Balance variables**	
mCTSIB	• Firm eyes open & eyes closed sway velocity (°/s) • Foam eyes open & eyes closed sway velocity (°/s) • Firm & foam Romberg ratio • Eyes open and eyes closed somatosensory ratio
Unilateral stance	• Eyes open and eyes closed left sway velocity (°/s) • Eyes open and eyes closed right sway velocity (°/s) • Composite eyes open & eyes closed sway velocity (°/s) • Symmetry angle eyes open & eyes closed (%)
Limits of stability	• Reaction time (s) • Movement velocity (°/s) • Endpoint excursion (%) • Maximum excursion (%) • Directional control (%)
Weight bearing squat	• Symmetry angle 0°, 30° & 60° (%)
**Gait variables**	
UGS and MGS trials	• Contact time (s) • Time to weight acceptance peak force (s) • Time to mid-stance peak force (s) • Time to push-off peak force (s) • Braking phase duration (s) • Propulsive phase duration (s) • Step frequency (Hz) • Weight acceptance peak force (BW) • Mid-stance peak force (BW) • Push-off peak force (BW) • Vertical peak force (BW) • Weight acceptance rate (BW/s) • Push-off rate (BW/s) • Braking peak force (BW) • Propulsion peak force (BW) • Braking force impulse (BWs) • Propulsive force impulse (BWs) • Change in horizontal velocity (m/s) • Step length index • Step width (m) • Heel strike ankle angle (°) • Heel strike knee angle (°) • Toe-off ankle angle (°) • Toe-off knee angle (°) • Mid-stance trunk angle (°) • Mid-stance knee angle (°)
	• Gait variability (MAD (%) for all the above)
**Strength variables**	
Isometric knee extension	• Peak torque (Nm/kg) • Symmetry angle (%) • RTD at 0-50ms, 0-100ms, 0-200ms (Nm/s/kg) • RTD symmetry angle at • 0-50ms, 0-100ms, 0-200ms (%)
Isokinetic knee flexion & extension (60°/s and 120°/s)	• Peak torque (Nm/kg) • Angle of peak torque (°) • Hamstrings/quadriceps ratio • Symmetry angle (%)
Isokinetic ankle dorsiflexion & plantar flexion (60°/s and 120°/s)	• Peak torque (Nm/kg) • Angle of peak torque (°) • Dorsiflexion/plantar flexion ratio • Symmetry angle (%)
Isokinetic trunk flexion & extension (20°/s and 45°/s)	• Peak torque (Nm/kg) • Angle of peak torque (°) • Flexion/extension ratio
**Body composition variables**	
Total body DXA	• Total fat mass (kg) • Percentage fat mass (%) • Fat mass index (kg/m^2^) • Fat mass symmetry angle (%) • Visceral adipose tissue (kg) • Total lean mass (kg) • Percentage lean mass (%) • Lean mass index (kg/m^2^) • Total appendicular lean mass (kg) • Percentage appendicular lean mass (%) • Appendicular lean mass index (kg/m^2^) • Appendicular lean mass index SA (%) • Upper-body appendicular lean mass (kg) • Lower-body appendicular lean mass (kg)
Left femur DXA	• Femoral neck BMD (g/cm^2^) • Upper neck BMD (g/cm^2^) • Lower neck BMD (g/cm^2^) • Ward’s triangle BMD (g/cm^2^) • Trochanter BMD (g/cm^2^) • Shaft BMD (g/cm^2^) • Total hip BMD (g/cm^2^) • Femoral neck T-score (SD) • Total hip T-score (SD)
Left femur DXA: Hip Structural Assessment	• Hip axis length (mm) • Femoral strength index • Buckling ratio • Section modulus (cm^3^) • Cross-sectional moment of inertia (mm^4^) • Cross-sectional area (mm^2^)
MQ	• MQ thigh (isometric knee extensor torque) • MQ thigh (knee extensor torque) • MQ thigh (combined torque) • MQ shank (plantar flexor torque) • MQ shank (combined torque)

BMD, bone mineral density; mCTSIB, Modified Clinical Test of Sensory Interaction on Balance; MGS, maximal gait speed; MQ, muscle quality; POMA, Performance Oriented Mobility Assessment; RTD, rate of torque development; TUG, Timed Up and Go; UGS, usual gait speed.

#### Balance data

The balance data were exported from the Balance Manager software, and any additional processing was completed using Microsoft Excel. In total, 52 variables were obtained from these protocols (Table 2). To quantify the visual contribution to balance during the firm and foam trials, the Romberg ratio (eyes closed sway velocity/eyes open sway velocity) was calculated [47]. To quantify the somatosensory contributions to balance during the eyes open and eyes closed trials, the somatosensory ratio (sway velocity foam/sway velocity firm) was calculated [48].

To determine inter-leg symmetry during the unilateral stance test and weight-bearing squat, the symmetry angle [49] was calculated for the eyes open and eyes closed trials, using the arctan function of the ratio of mean values measured from the left and right limbs [50]. As the focus of this analysis was to evaluate the magnitude of asymmetry rather than the direction, the absolute values were reported.

#### Gait data

GRF data processing was completed using BioWare software (version 5.3.1.7; Kistler Instruments Ltd., Winterthur, Switzerland), and the video files were analysed using Simi Motion analysis (version 9.2.3; SIMI Reality Motion Systems, Munich, Germany). Overall, 99 variables (Table 2) were processed from the gait data and included in further analysis. Five successful steps for both gait speeds were identified per participant and included within the analysis. For each participant, the five steps analysed were from the same limb. The GRF data were filtered with a second-order low pass Butterworth filter, with a 50 Hz cut-off frequency [51]. UGS and MGS (m/s) were determined for each trial using the distance walked and ambulation time measured from the timing gates. Gait speed reserve was calculated as the ratio between MGS and UGS [52], and was used to quantify the capacity to increase walking speed when needed (as noted above, UGS, MGS and gait speed reserve were analysed in the clinical measures dataset).

Gait variability was measured using the median absolute deviation (MAD) using the five steps for each variable. The MAD provides a robust estimate of variability, which is less sensitive to outliers and artificial inflation compared with other measures such as the coefficient of variation [53]. To allow for comparisons between groups and variables, the MAD scores for each variable were reported as a percentage of the original median value [54]. The percentage MAD scores were calculated for all gait variables at both gait speeds, apart from step width and change in horizontal velocity.

#### Strength data

In total, 70 strength variables (Table 2) were obtained from the different protocols and included in further analysis. The peak torque trials (sampled at 100 Hz) were exported from the Biodex software for processing using in-house algorithms written in MATLAB (The MathWorks Inc., Natick, Massachusetts, USA). Flexion/extension strength ratios were calculated for the trunk, knee and ankle by taking the quotients between the isokinetic flexor peak torque and isokinetic extensor peak torque. These were calculated for all testing speeds and were determined for the left and right limbs during the ankle and knee trials. For the RTD trials, the torque signal from the dynamometer was sampled at 2000 Hz using a Biopac MP150 data acquisition system integrated with the AcqKnowledge software (version 4.4; Biopac Systems Inc., Santa Barbara, CA, USA). The signal was exported and processed offline using custom written algorithms in MATLAB (The MathWorks Inc., Natick, Massachusetts, USA). The signal was filtered using a second-order low-pass Butterworth filter, with a cut-off frequency of 150 Hz [55]. RTD was defined as the slope of the torque-time curve (△torque/△time) for three time intervals from the onset of the contraction [56]. The onset was determined as the point where the torque signal reached 4 Nm above baseline [57]. These time periods were chosen to represent the rapid muscle responses (≤200 ms) needed to prevent falling when regaining balance following a trip or slip incident [58]. To determine inter-leg symmetry for the peak torque values, the symmetry angle (%) was calculated using the methods described previously.

#### Body composition data

All data from both DXA scans were analysed using the enCORE software (version 15.0; GE Healthcare, Madison, WI). For the body composition analysis, regions of interest and cut points were automatically determined by the enCORE software. A total of 14 body composition variables, nine BMD variables, six hip structure variables, and five MQ variables were obtained and included in further analysis (Table 2). Several variables were estimated from the total body scan and additional regions of interest were manually segmented to estimate lean tissue mass in the upper and lower leg for both limbs.

MQ was defined as muscle strength per kilogram of lean tissue mass [59]. Upper leg MQ was calculated using the following equations: isometric knee extension peak torque/upper leg lean tissue mass, isokinetic (60°/s) knee extension peak torque/upper leg lean tissue mass, and isokinetic (60°/s) combined peak torque (knee extension + flexion)/upper leg lean tissue mass [59]. Lower leg MQ was defined using the following equations: isokinetic (60°/s) plantar flexion peak torque/lower leg lean tissue mass, and isokinetic (60°/s) combined peak torque (plantar flexion + dorsiflexion)/lower leg lean tissue mass [60]. These indices were defined for the dominant and non-dominant limbs, based on the highest torque measured during the left and right trials. In addition, a composite measure of MQ was defined for each of the five indices, which was an average measure of the dominant and non-dominant limbs. Inter-leg symmetry was calculated for each of the MQ indices using the symmetry angle (%) as described previously.

### Statistical analyses

In total, 281 variables were included in the analysis. Because the number of variables was much greater than the number of participants (n = 60), it was not possible to use techniques like logistic regression. Such techniques utilise the generalised linear model, which would have resulted in instability problems in this scenario [16]. Therefore, we developed an analysis strategy that could cope with the size and complexity of the data set together with its inherent multicollinearity. The data were initially partitioned into five single-domain sub-datasets, hereafter referred to as ‘data packages’ (i.e. the balance, gait, clinical, strength and body composition data packages), thus making the analysis more manageable. The data in each of the single domains were analysed to determine the relative importance and discriminatory ability of variables when differentiating between fallers and non-fallers. A consistent analysis strategy was used across all data packages. Following this, the strongest discriminators from each single-domain analysis were compiled into a final multi-domain dataset and were analysed together to identify which variables collectively best discriminated between the two cohorts. All analyses were undertaken using in-house algorithms and code written in R [61].

#### Single-domain analysis

The initial single-domain results were derived using traditional univariate techniques before applying a machine learning multivariate approach. Firstly, two-sample t-tests and effect sizes (Cohen’s *d*) were used to determine differences between fallers and non-fallers with statistical significance set at *p*<0.05. For categorical variables, group differences and effect sizes were determined using chi-squared tests and Cramer’s V. Pearson’s correlation coefficients were also used to quantify the relationships between variables, with *r* values of 0.10, 0.30 and 0.50 representing small, moderate and large associations, respectively [62]. The absolute value of the correlation coefficient was also used to provide an indication of multicollinearity between variables, with *r* values >0.50 suggesting high collinearity [63]. Bartlett’s test of sphericity was used to assess the redundancy in the data using the correlation matrix [64]. This test determined whether the correlation matrix was significantly different from an identity matrix (i.e. a matrix with ones along the diagonal and zeros for all other entries). From Bartlett’s test, *p*<0.05 indicated that there was redundancy in the data and that the data were not orthogonal (i.e. uncorrelated).

From a machine learning perspective, random forest analysis (based on 500 trees) [65]) and leave-one-variable-out (LOVO) partial least squares correlation analysis (PLSCA; as described by Weaving et al. 2019 [16]) were employed to identify the important variables that best discriminated between fallers and non-fallers. Variables were identified as important if they were above the cut-off ‘elbow’ on the variable importance scree plots produced using both techniques. A mixed-methods approach, using the Jenks natural breaks algorithm [66] with a subjective validation, was used to determine the optimal cut-off point for important variables. Following this, the important variables selected from both techniques were combined into two refined datasets (comprising a smaller number of variables). Using the refined datasets, classification models were constructed to differentiate between fallers and non-fallers, using PLSCA [67], random forest [65], and logistic regression techniques. Further details about the random forest, PLSCA and logistic regression techniques are presented in S1 Appendix.

The receiver operating characteristics (ROC) area under the curve (AUC) was used throughout as a metric of diagnostic accuracy, providing an index of discriminatory ability [68]. An AUC value of 0.50 represented no discriminatory ability, with values of 0.70 to 0.79 considered acceptable, 0.80 to 0.89 considered excellent, and ≥0.90 considered outstanding [68]. An AUC value of one represents perfect discrimination between groups.

#### Multi-domain analysis

Overall, 51 variables were included in the further multi-domain analysis. These variables were identified as important by the random forest and LOVO PLSCA during the five single-domain analyses. When compiling the multi-domain data package, some participants had missing values. Therefore, for the multi-domain analysis it was necessary to impute the missing data so that all observations were included (n = 60; fallers = 21, non-fallers = 39), thus enabling the use of the same consistent machine learning strategy and minimising the loss of valuable information that could have been beneficial for discrimination between groups. As such, any missing values were imputed using the Probabilistic Principal Component Analysis (PPCA) technique [69].

Analysis was undertaken using the same multivariate machine learning approach described for the single domains. Following traditional univariate analyses, random forest and LOVO PLSCA were employed to identify the important variables which best discriminated between fallers and non-fallers, as well as highlighting variables of less importance. Subsequently, the important variables selected from both techniques (i.e. above the cut-off elbow on the variable importance plots) were combined into one final refined dataset. Using this refined dataset, classification models were constructed to differentiate between fallers and non-fallers, using PLSCA, random forest and logistic regression techniques.

To determine the general applicability of the classification models (i.e. to test how each model performed on unseen data) and to check that the models were not overfitting the data, cross validation was performed. For the random forest models, the inherent out-of-bag cross validation methods were used (meaning that a separate cross validation was not needed) [70]. Following pilot work, leave-one-out cross validation (LOOCV) was deemed most appropriate for the logistic regression models [71]. This is supported by previous work which has suggested that LOOCV performs well with small sample sizes and produces comparable results to 10-fold cross validation methods [72]. Further details about the cross validation are presented in S1 Appendix.

In addition, and for the first time in this area, PLSCA was used to quantify the strength of the relationships between the various domains. Whilst univariate correlation analyses were performed to quantify the associations between individual variables in each data package, PLSCA has the advantage that it can quantify the strength of the relationships between multiple groups of variables in different domains. This was done by applying PLSCA bilaterally between the domains. The amount of shared information was determined by calculating the singular value inertia, with greater values representing more shared information and stronger relationships between the single-domain data packages [73].

## Results

### Single-domain analysis

The full data and univariate results for the single-domain analyses are presented in S1–S9 Tables. The important variables identified in each data package were included in single-domain classification models to differentiate between fallers and non-fallers, using PLSCA, random forest and logistic regression techniques.

The range of ROC results for all the single-domain classification models (PLSCA, random forest and logistic regression) are shown in Table 3. It was possible to discriminate between fallers and non-fallers with an AUC≥0.90 in the gait and balance data packages and an AUC≥0.80 in the strength data package. The clinical measures and body composition data packages demonstrated acceptable discriminatory ability (AUC≥0.70) between groups.

**Table 3:** Results of the ROC analyses using the variables in the single-domain analyses.

Data package (number of models*)	AUC	Sensitivity	Specificity	*p* value
Balance data package (10 models)	0.67 to 0.94	73% to 93%	56% to 88%	All <0.001
Gait data package (7 models)	0.84 to 0.97	76% to 94%	82% to 92%	All <0.001
Clinical measures data package (8 models)	0.58 to 0.77	67% to 83%	44% to 79%	All <0.001
Strength data package (7 models)	0.61 to 0.86	58% to 83%	59% to 93%	<0.001 to 0.02
Body composition data package (7 models)	0.56 to 0.78	50% to 92%	46% to 82%	<0.001 to 0.17

AUC, area under the curve.

* In each of the single-domain analyses, baseline and refined models were produced using each of the PLSCA, random forest, and logistic regression techniques. In each single domain, the baseline models included all variables in the data package. These baseline models were then refined using only the variables identified as important by firstly the random forest and secondly the LOVO PLSCA. Therefore, several models were produced for each data package in the single-domain analyses. The total number of models differed depending on the number of refined logistic regression models produced.

### Multi-domain analysis

51 variables identified as important from the five single-domain analyses were included in the multi-domain data package. The balance, gait and clinical measures data included in the multi-domain analysis are presented in Table 4. From the balance data, significant differences were observed between fallers and non-fallers for the variables right directional control (*p* = 0.03, Cohen’s *d* = 0.56) and anterior maximum excursion (*p* = 0.04, Cohen’s *d* = 0.60). Fallers demonstrated greater right directional control (7%) and less anterior maximum excursion (11%) compared with non-fallers. From the gait data, significant differences were reported for all variables apart from MAD UGS step length (*p* = 0.18, Cohen’s *d* = 0.47). Fallers walked with shorter steps (UGS = 7%, MGS = 6%), a greater degree of knee flexion at toe-off (UGS = 2%, MGS = 1%), and a longer braking phase (7%) compared with non-fallers. In terms of gait variability, fallers demonstrated less variability for MGS step frequency (44%) and UGS braking phase duration (44%), alongside greater variability for MGS toe-off knee angle (41%), UGS braking peak force (40%), and UGS time to mid-stance peak force (46%) compared with non-fallers. For the clinical measures data, significant differences were observed between fallers and non-fallers for TUG time, Tinetti POMA total score, and UGS. Fallers had significantly slower TUG time and UGS compared with non-fallers (8% and 5% respectively), alongside a smaller Tinetti POMA total score (4%).

**Table 4:** Descriptive statistics for the important balance, gait and clinical measures variables included in the multi-domain analysis for fallers (n = 21) and non-fallers (n = 39).

	Fallers	Non-fallers	*p* value	Effect size
**Balance variables**				
Posterior endpoint excursion (%)	43.95±13.34	42.62±12.02	0.71	0.11
Anterior maximum excursion (%)	82.05±17.60	91.67±15.52	0.04**	0.60
Eyes closed left sway velocity (°/s)	5.67±3.18	6.03±2.71	0.67	0.12
Posterior directional control (%)	65.52±20.38	59.35±19.08	0.26	0.32
Right endpoint excursion (%)	61.57±22.52	65.87±15.82	0.44	0.23
Eyes closed right sway velocity (°/s)	7.02±3.56	5.97±2.75	0.25	0.34
Right directional control (%)	81.1±8.28	75.74±10.28	0.03**	0.56
Eyes open right sway velocity (°/s)	1.61±0.98	1.24±0.45	0.12	0.54
Eyes closed somatosensory ratio	4.07±1.35	3.93±1.38	0.70	0.10
Composite directional control (%)	78.27±7.36	74.25±8.11	0.06*	0.51
Foam eyes closed sway velocity (°/s)	1.62±0.45	1.49±0.43	0.29	0.30
**Gait variables**				
UGS braking phase duration (s)	0.32±0.03	0.30±0.03	0.02**	0.65
UGS TO knee angle (°)	127±4	130±5	0.01**	0.75
UGS step length index	0.43±0.02	0.46±0.03	<0.001***	1.03
MAD UGS step length (%)	1.80±1.38	1.37±0.47	0.18	0.47
MAD UGS braking phase duration (%)	2.77±1.85	4.94±2.64	<0.001***	0.91
MGS step length index	0.49±0.02	0.52±0.03	<0.001***	0.93
MAD MGS TO knee angle (%)	1.17±0.54	0.83±0.47	0.02**	0.68
MAD MGS step frequency (%)	0.62±0.52	1.11±0.82	0.01**	0.67
MGS TO knee angle (°)	132±3	134±4	0.04**	0.53
MAD UGS Breaking Peak Force (%)	6.45±3.83	4.62±2.24	0.05**	0.63
MAD UGS Time to Mid-stance Peak Force (%)	2.59±1.72	1.78±0.88	0.05**	0.65
**Clinical measures variables**				
POMA total score	26.22±1.7	27.31±1.17	0.01**	0.79
TUG time (s)	8.24±1.18	7.61±1.17	0.05**	0.54
POMA balance score	15.09±1.58	15.69±0.57	0.10*	0.59
UGS (m/s)	1.42±0.12	1.50±0.14	0.04**	0.54
GS reserve	1.33±0.13	1.29±0.12	0.29	0.30

UGS, usual gait speed; TO, toe-off; MAD, median absolute deviation; MGS, maximal gait speed.

Data are presented as mean ± SD. Group differences and effect sizes were determined using two-tailed t-tests and Cohen’s *d*.

* *p≤*0.10

** *p≤*0.05

*** *p≤*0.001.

For the strength data included in the multi-domain analysis (Table 5), significant differences were reported for dorsiflexion 120 dominant peak torque, knee flexion 120 dominant peak torque, knee flexion 120 non-dominant peak torque, knee flexion 60 non-dominant peak torque, plantar flexion 60 non-dominant peak torque, knee flexion 120 symmetry angle, knee flexion 60 dominant peak torque, dorsiflexion 60 non-dominant peak torque, and dorsiflexion 120 non-dominant peak torque. Fallers produced lower peak torque compared with non-fallers. For knee flexion 120 symmetry angle, fallers demonstrated greater asymmetry compared with non-fallers. For the body composition data (Table 5), fallers demonstrated lower thigh MQ (combined torque) compared with non-fallers (*p* = 0.04, Cohen’s *d* = 0.54).

**Table 5:** Descriptive statistics for the important strength and body composition measures include in the multi-domain analysis for fallers (n = 21) and non-fallers (n = 39).

	Fallers	Non-fallers	*p* value	Effect size
**Strength variables**				
Plantar flexion 120 DL PT (Nm/kg)	0.43±0.16	0.51±0.15	0.06*	0.54
Plantar flexion 60 SA (%)	7.77±6.51	6.68±4.48	0.50	0.21
Knee extension 60 SA (%)	4.59±4.41	4.55±3.46	0.98	0.01
Dorsiflexion 120 DL PT (Nm/kg)	0.20±0.04	0.23±0.05	0.01**	0.71
Plantar flexion 120 NDL PT (Nm/kg)	0.34±0.13	0.41±0.13	0.06*	0.53
Knee flexion 120 DL PT (Nm/kg)	0.59±0.15	0.67±0.15	0.05**	0.54
Knee flexion 120 NDL PT (Nm/kg)	0.49±0.15	0.59±0.15	0.02**	0.68
Knee flexion 60 NDL PT (Nm/kg)	0.63±0.13	0.71±0.14	0.04**	0.58
Plantar flexion 60 NDL PT (Nm/kg)	0.50±0.17	0.60±0.22	0.05**	0.50
Knee flexion 120 SA (%)	6.72±4.41	4.33±2.44	0.03**	0.73
Trunk flexion 45 AngPT (°)	57.62±11.64	51.98±9.37	0.07*	0.55
Knee flexion 60 DL PT (Nm/kg)	0.74±0.12	0.81±0.13	0.04**	0.56
Dorsiflexion 60 NDL PT (Nm/kg)	0.23±0.04	0.27±0.06	0.01**	0.67
Dorsiflexion 120 NDL PT (Nm/kg)	0.17±0.04	0.20±0.05	0.01**	0.64
**Body composition variables**				
DL Thigh MQ (combined torque; Nm/kg)	34.01±4.26	36.07±5.18	0.10*	0.42
SA shank MQ (PF torque; Nm/kg)	7.48±5.40	7.27±4.87	0.88	0.04
DL thigh MQ (KE torque; Nm/kg)	21.98±3.12	23.43±3.92	0.12	0.40
Thigh MQ (combined torque; Nm/kg)	31.69±4.73	34.44±5.22	0.04**	0.54
Femoral strength index	1.37±0.29	1.45±0.23	0.28	0.32
DL thigh MQ (Isometric KE torque; Nm/kg)	30.09±5.00	32.21±7.02	0.18	0.33
Trochanter BMD (g/cm^2^)	0.70±0.11	0.67±0.10	0.27	0.31
NDL shank MQ (PF torque; Nm/kg)	19.66±6.85	22.34±8.53	0.19	0.34
ALMI (kg/m^2^)	6.27±0.72	6.21±0.68	0.76	0.08
Total fat mass (kg)	24.14±7.69	21.46±7.19	0.20	0.36

ALMI, appendicular lean mass index; AngPT, angle of PT; BMD, bone mineral density; DL, dominant limb; FM, fat mass; FMI, fat mass index; KE, knee extension; MQ, muscle quality; NDL, non-dominant limb; PF, plantar flexion; PT, peak torque; SA, symmetry angle; VAT, visceral adipose tissue.

Data are presented as mean ± SD. Group differences and effect sizes were determined using two-tailed t-tests and Cohen’s *d*.

* *p≤*0.10

** *p≤*0.05

*** *p≤*0.001.

**Fig 2:**
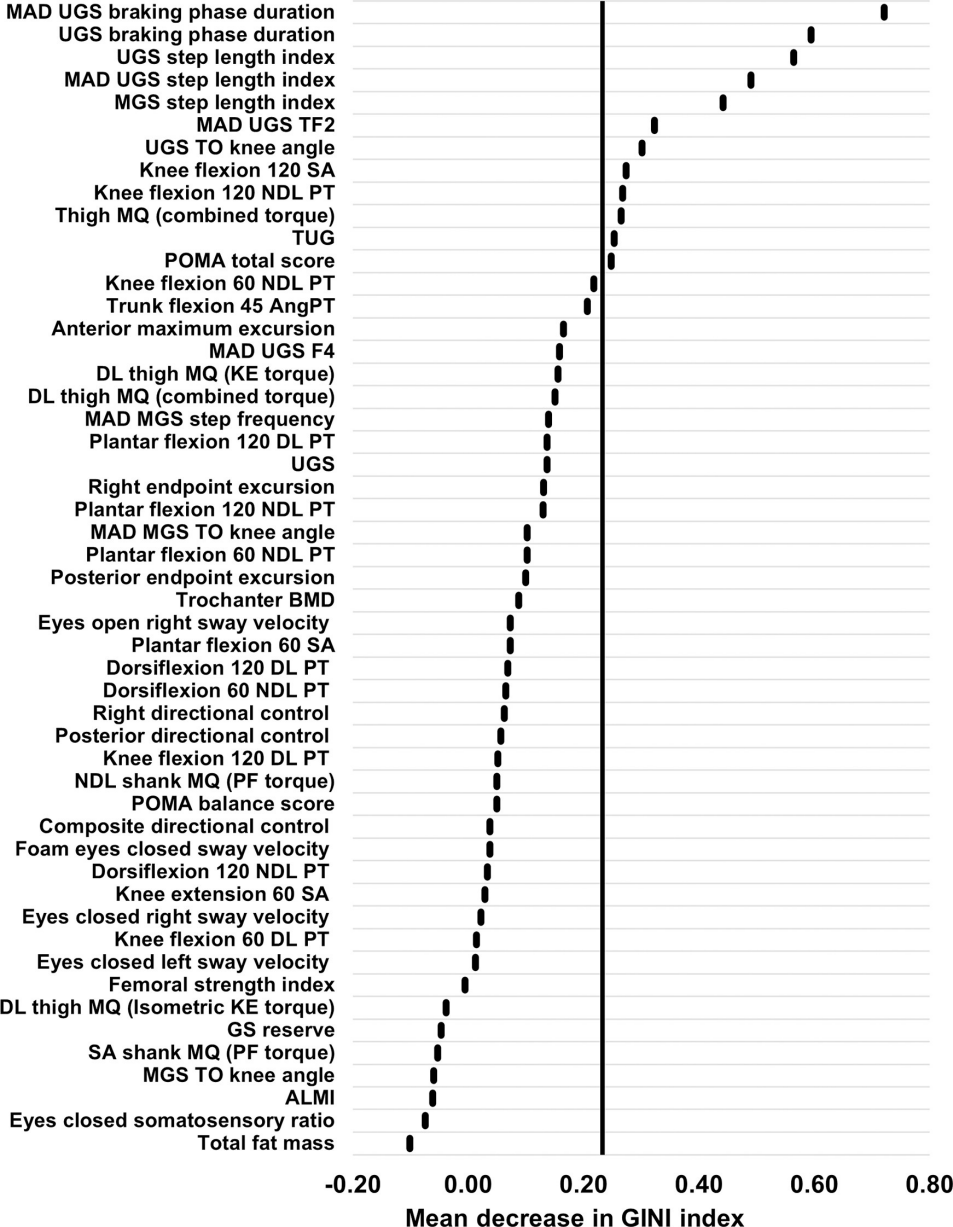
Variable importance plot from the random forest. This highlights the mean decrease in the Gini index attributable to each predictor variable in the multi-domain data package. Variables to the right of the line (n = 12) were shown to be important and were included within the refined dataset. ALMI, appendicular lean mass index; AngPT, angle of peak torque; BMD, bone mineral density; DL, dominant limb; F4, braking peak force; GS, gait speed; KE, knee extension; MAD, median absolute deviation; MGS, maximal gait speed; MQ, muscle quality; NDL, non-dominant limb; PF, plantar flexion; PT, peak torque; POMA, Performance Oriented Mobility Assessment; SA, symmetry angle; TF2, time to mid-stance peak force; TO, toe-off; TUG, Timed Up and Go; UGS, usual gait speed.

**Fig 3:**
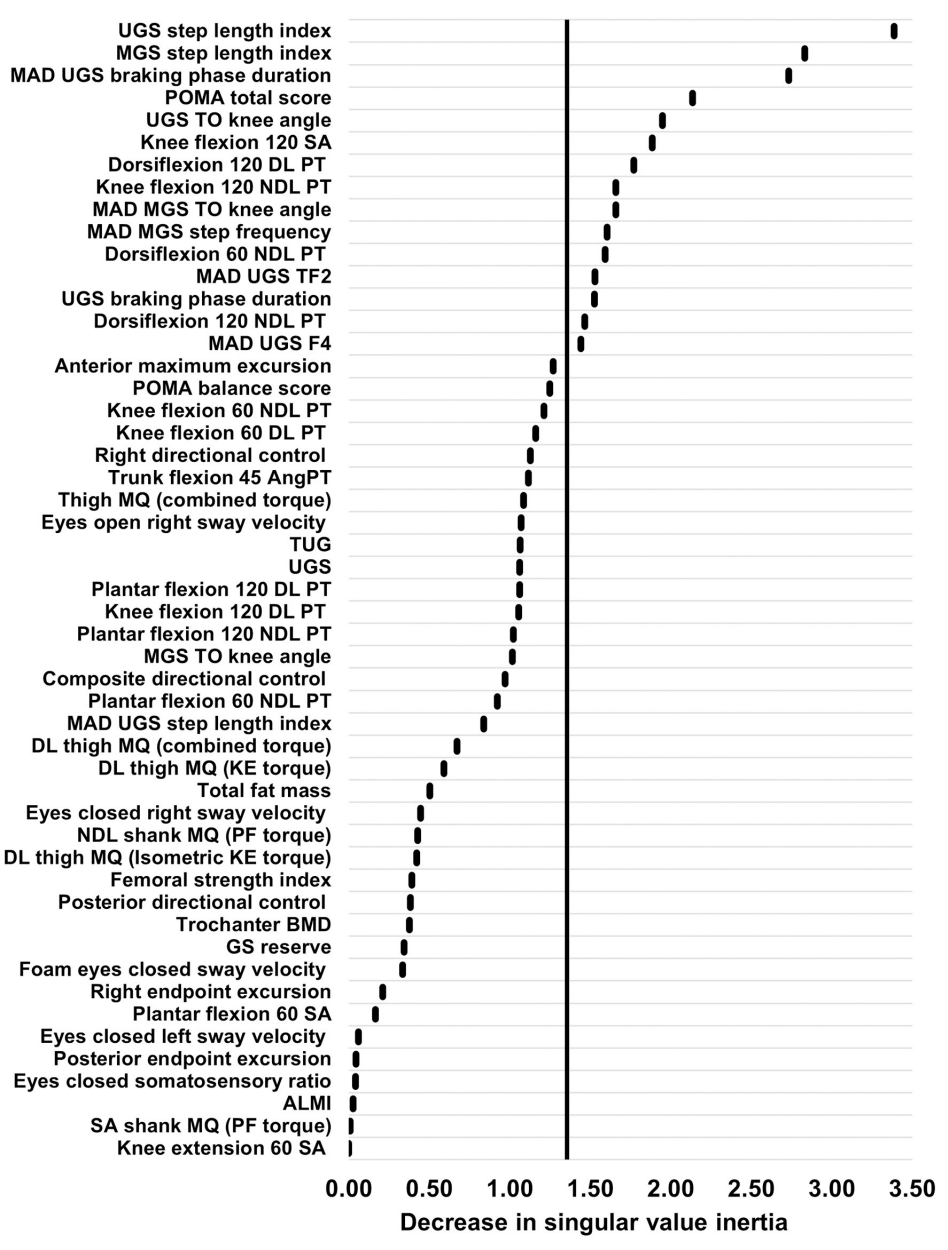
Variable importance plot from the LOVO PLSCA. This highlights the decrease in singular value inertia attributable to each predictor variable in the multi-domain data package. Variables to the right of the line (n = 15) were shown to be important and were included within the refined dataset. ALMI, appendicular lean mass index; AngPT, angle of peak torque; BMD, bone mineral density; DL, dominant limb; F4, braking peak force; GS, gait speed; KE, knee extension; MAD, median absolute deviation; MGS, maximal gait speed; MQ, muscle quality; NDL, non-dominant limb; PF, plantar flexion; PT, peak torque; POMA, Performance Oriented Mobility Assessment; SA, symmetry angle; TF2, time to mid-stance peak force; TO, toe-off; TUG, Timed Up and Go; UGS, usual gait speed.

Random forest analysis and LOVO PLSCA were used to quantify the relative importance of the variables in the imputed multi-domain data package with respect to their ability to discriminate between fallers and non-fallers. Using the random forest analysis, 12 variables had the greatest ability to distinguish between fallers and non-fallers (Fig 2). From the selected variables, seven were from the gait data, two were from the strength data, two were from the clinical measures data, and one was from the body composition data. Using the LOVO PLSCA, 15 variables had the greatest ability to distinguish between fallers and non-fallers (Fig 3). From the selected variables, nine were from the gait data, five were from the strength data, and one was from the clinical measures data.

Although the random forest and LOVO PLSCA ranked the variables in different orders of importance, both techniques identified the following nine variables as important: MAD UGS braking phase duration, UGS braking phase duration, UGS step length index, MGS step length index, MAD UGS time to mid-stance peak force, UGS toe-off knee angle, knee flexion 120 symmetry angle, knee flexion 120 non-dominant limb peak torque, and Tinetti POMA total score.

Following these analyses, the selected ‘important’ variables from both techniques were combined into a refined multi-domain dataset (Table 6). Overall, 18 important variables were included, with ten variables from the gait data, five from the strength data, two from the clinical measures data, and one from the body composition data. It should be noted that, rather surprisingly, no variables were selected as important from the balance data. This refined dataset was then used when constructing optimal classification models to differentiate between fallers and non-fallers, using PLSCA, random forest and logistic regression techniques.

**Table 6:** Outline of the refined multi-domain dataset.

Selected variables in the refined multi-domain dataset*
• **MAD UGS braking phase duration** • **UGS braking phase duration** • **UGS step length index** • MAD UGS step length • **MGS step length index** • **MAD UGS time to mid-stance PF** • **UGS TO knee angle** • **KF 120 SA** • **KF 120 NDL PT** • Thigh MQ (combined torque) • TUG • **POMA total score** • DF 120 DL PT • MAD MGS TO knee angle • MAD MGS step frequency • DF 60 NDL PT • DF 120 NDL PT • MAD UGS braking force

DL, dominant limb; KF, knee flexion; MAD, median absolute deviation; MGS, maximal gait speed; MQ, muscle quality; NDL, non-dominant limb; PF, plantar flexion; PT, peak torque; POMA, Performance Oriented Mobility Assessment; SA, symmetry angle; TO, toe-off; TUG, Timed Up and Go; UGS, usual gait speed.

* The nine variables identified as important using both the random forest and LOVO PLSCA techniques are highlighted in bold.

The results of the ROC analyses for the classification models using the refined multi-domain dataset are presented in Table 7. The PLSCA model (inertia = 81.74, *p*<0.001) demonstrated an excellent ability to distinguish between fallers and non-fallers (AUC≥0.80), with 18/21 fallers (sensitivity = 86%) and 28/39 non-fallers (specificity = 72%) classified correctly. The random forest model (out-of-bag error rate = 27%, *p* = 0.11) demonstrated an acceptable ability (AUC>0.70) to discriminate between fallers and non-fallers, with 17/21 fallers (sensitivity = 81%) and 29/39 non-fallers (specificity = 74%) classified correctly.

**Table 7:** Results of the ROC analysis using the variables in the refined multi-domain data package.

Model	Cut-off threshold	AUC	Sensitivity	Specificity	*p* value
PLSCA	1.37	0.84	86%	72%	<0.001
Random forest	0.34	0.75	81%	74%	<0.001
Logistic regression	0.35	0.92	90%	87%	<0.001
Refined logistic regression	0.35	0.89	81%	85%	<0.001

AUC, area under the curve.

For the logistic regression analysis, two models were constructed. The first model included all 18 important variables, with a LOOCV accuracy of 63% (Table 8). This model demonstrated an outstanding ability to distinguish between fallers and non-fallers (AUC≥0.90), with 19/21 fallers (sensitivity = 90%) and 34/39 non-fallers (specificity = 87%) classified correctly (Table 7). However, most of the predictor variables failed to reach significance, suggesting that this model was likely to be over-fitted to the data.

**Table 8:** Baseline logistic regression model using the important predictors in the refined multi-domain dataset.

Response variable	Predictor variables	Coefficient (b)	*p* value	VIF
Falls History	Intercept	28.46	0.12	NA
	MAD UGS braking phase duration	-0.39	0.15	2.24
	UGS braking phase duration	-8.72	0.70	3.26
	UGS step length index	0.13	0.98	8.13
	MAD UGS step length	0.43	0.54	2.51
	MGS step length index	-8.29	0.85	10.57
	MAD UGS TF2	0.57	0.15	1.74
	UGS TO knee angle	-0.21	0.11	1.52
	KF 120 SA	0.29	0.15	2.93
	KF 120 NDL PT	4.96	0.52	8.98
	Thigh MQ (combined torque)	-0.09	0.59	5.52
	TUG	-0.08	0.86	1.87
	POMA total score	0.20	0.59	2.67
	DF 120 DL PT	-7.05	0.84	17.24
	MAD MGS TO knee angle	0.66	0.55	1.57
	MAD MGS step frequency	-1.82	0.10*	2.93
	DF 60 NDL PT	19.22	0.46	10.21
	DF 120 NDL PT	-24.11	0.51	17.81
	MAD UGS F4	0.04	0.86	3.00

AIC; Akaike’s information criterion; BIC, Bayesian information criterion; DF, dorsiflexion; F4, braking peak force; KF, knee flexion; MAD, median absolute deviation; MGS, maximal gait speed; MQ, muscle quality; NDL, non-dominant limb; POMA, Performance Oriented Mobility Assessment; PT, peak torque; SA, symmetry angle; TF2, time to mid-stance peak force; TO, toe-off; TUG, timed up and go; UGS, usual gait speed; VIF, variable inflation factor.

AIC = 80.36, BIC = 120.15, McFadden R^2^ = 0.45.

* *p≤*0.10

** *p≤*0.05

*** *p≤*0.001.

**Fig 4:**
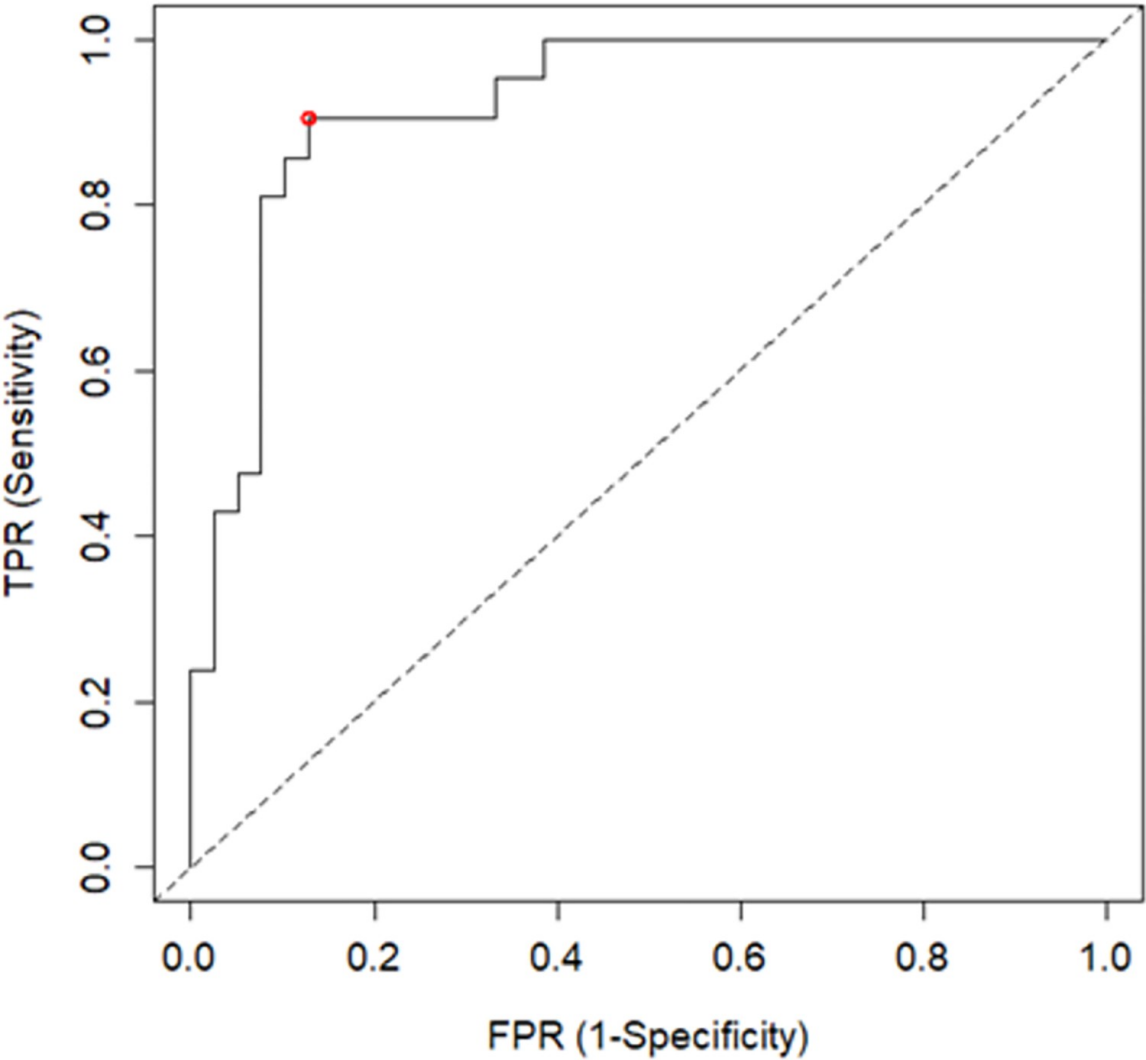
ROC curve for the multi-domain model. The optimal cut-off threshold is highlighted with the red circle. FPR, false positive rate; TPR, true positive rate.

To improve model fit, the logistic regression model was refined using multiple approaches. Firstly, backwards exclusion was performed by hand and by using an automatic step function based on minimizing the Akaike information criterion. Following this, the model was also refined based on minimizing the Bayesian information criterion. Overall, these approaches resulted in the same refined model (Table 9). This refined model (Fig 4) reached significance, with a LOOCV accuracy of 75%. The refined model demonstrated an excellent ability (AUC≥0.80) to differentiate between groups, with 17/21 fallers (sensitivity = 81%) and 33/39 non-fallers (specificity = 85%) classified correctly (Table 9).

**Table 9:** Refined logistic regression model using the important predictors in the multi-domain data package.

Response variable	Predictor variables	Coefficient (b)	*p* value	VIF
Falls History	Intercept	30.00	0.03**	NA
	MAD UGS Braking Phase Duration	-0.47	0.02**	1.09
	MAD UGS TF2	0.53	0.06*	1.07
	UGS TO Knee Angle	-0.24	0.03**	1.16
	KF 120 SA	0.22	0.09*	1.04
	MAD MGS step frequency	-1.02	0.08*	1.06

AIC; Akaike’s information criterion; BIC, Bayesian information criterion; KF, knee flexion; MAD, median absolute deviation; SA, symmetry angle; TF2, time to mid-stance peak force; TO, toe-off

UGS, usual gait speed; VIF, variable inflation factor.

AIC = 58.39, BIC = 70.96, McFadden R^2^ = 0.40.

* *p≤*0.10

** *p≤*0.05

*** *p≤*0.001.

### Relationship between domains

**Fig 5:**
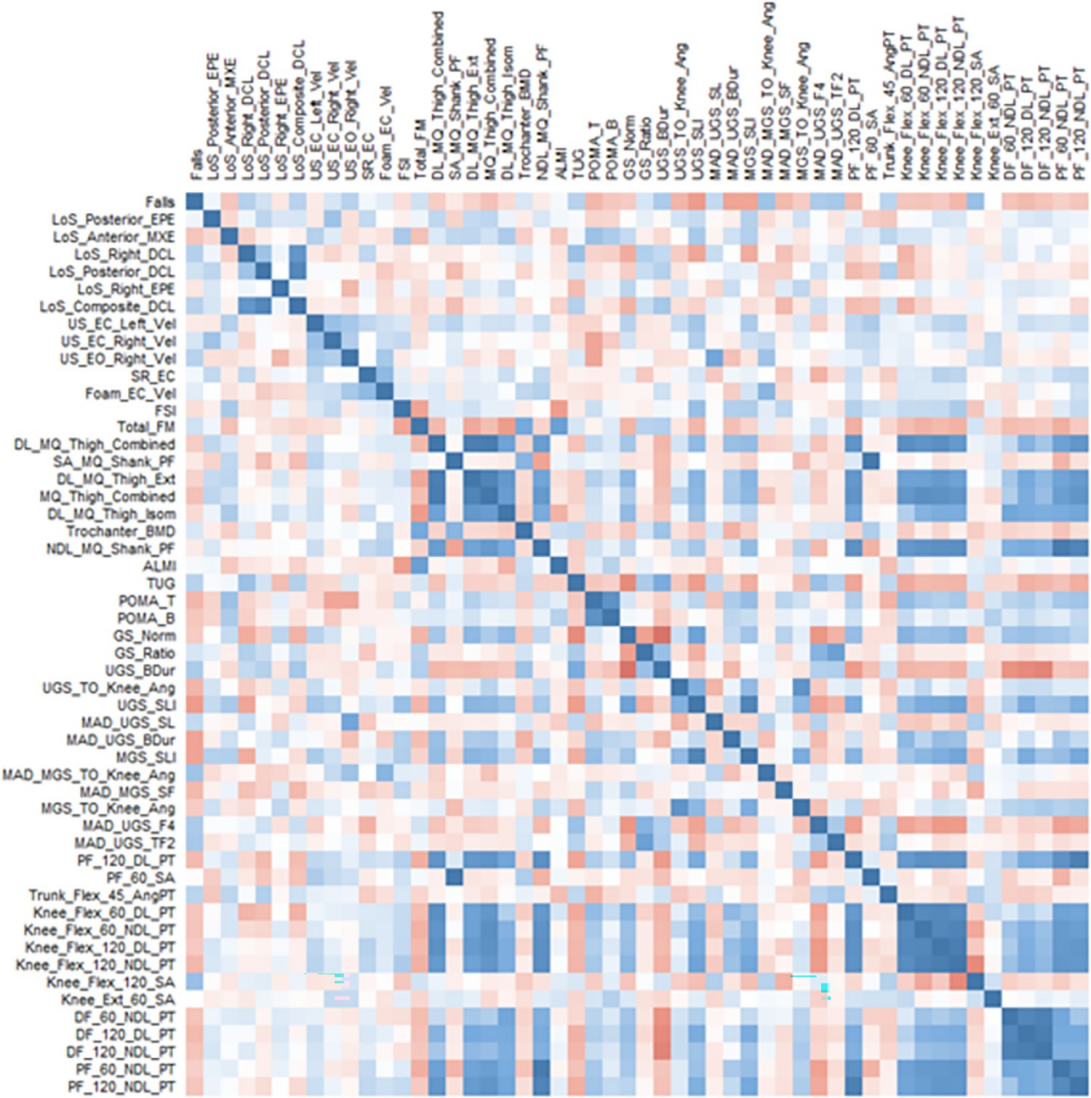
Correlation matrix heatmap with Pearson’s r coefficients colour coded for the multi-domain analysis. Stronger relationships (positive or negative) are represented in a darker colour. ALMI, appendicular lean mass index; Ang, angle; AngPT, angle of peak torque; BDur, braking phase duration; BMD, bone mineral density; DCL, directional control; DF, dorsiflexion; DL, dominant limb; EC, eyes closed; EO, eyes open; EPE, endpoint excursion; Ext, extension; F4, braking peak force; Flex, flexion; FM, fat mass; FSI, Femoral Strength Index; GS_Norm, usual gait speed; GS_Ratio, gait speed reserve; Isom, isometric; LOS, limits of stability; MAD, median absolute deviation; MGS, maximal gait speed; MQ, muscle quality; MXE, maximum excursion; NDL, non-dominant limb; PF, plantar flexion; POMA, Performance Oriented Mobility Assessment; POMA_B, POMA balance score; POMA_T, POMA total score; PT, peak torque; SA, symmetry angle; SF, step frequency; SL, step length; SLI, step length index; SR, somatosensory ratio; TF2, time to mid-stance peak force; TO, toe-off; TUG, Timed Up and Go; UGS; usual gait speed; US, unilateral stance; Vel, sway velocity.

Pearson correlation analyses revealed multiple associations between the variables within the multi-domain data package, as shown in the heatmap presented in Fig 5. These results suggest that there is a degree of multicollinearity present within this data package (particularly within the body composition and strength domains, where the within-group correlations are very strong) and that some of the variables are not independent of each other (i.e. they are strongly correlated). Of note are the between-domain relationships within the multi-domain data package. Interestingly, there are much weaker associations between the balance variables and those in the other domains, with the strongest relationship reported between foam eyes closed sway velocity, and MAD MGS toe-off knee angle having only a moderate effect size (r = 0.45, *p*<0.001). By comparison, much stronger relationships were exhibited between the body composition and strength domains, with, for example, the correlation between non-dominant shank MQ (plantar flexion torque) and plantar flexion 60 non-dominant peak torque being r = 0.92 (*p*<0.001). There were also moderately strong significant relationships observed between the gait variables and strength variables, with, for example, the correlation between UGS step length index and knee flexion 120 non-dominant peak torque being r = 0.58 (*p*<0.001).

PLSCA was used to quantify the strength of the relationships (i.e. shared information) between the groups of variables in each single domain. The results of these analyses are presented in Table 10. In line with the correlation results, the strongest relationships (i.e. highest normalised inertia) were identified between the strength and body composition data packages (normalised inertia: 19.35), and the strength and gait data packages (normalised inertia: 14.11). Interestingly, the weakest relationships (i.e. lowest inertia) were shown between the body composition and clinical measures data packages (normalised inertia: 8.25), and the clinical measures and the strength data packages (normalised inertia: 9.00).

**Table 10:** PLSCA between variables from different domains.

Variables	Balance	Body Composition	Clinical Measures	Gait	Strength
**Balance**	**NA**	206.61	166.08	263.55	241.39
		9.84	10.38	11.98	9.66
		0.07*	<0.001***	<0.001***	0.10*
**Body Composition**		**NA**	123.74	241.16	464.28
			8.25	11.48	19.35
			0.04**	<0.001***	<0.001***
**Clinical Measures**			**NA**	195.55	170.94
				12.22	9.00
				<0.001***	0.001***
**Gait**				**NA**	352.62
					14.11
					<0.001***
**Strength**					**NA**

NA, not applicable.

Data are presented as measured inertia, normalised inertia, and *p* value.

* *p≤*0.10

** *p≤*0.05

*** *p≤*0.001.

## Discussion

The aims of this study were to: a) identify the functional and physical factors that can best differentiate between fallers and non-fallers in older women; and b) quantify the relative importance of these variables. The findings demonstrate that it is possible to discriminate between fallers and non-fallers with a high degree of accuracy using a refined set of 18 variables drawn from four domains, with the gait and strength domains being particularly informative for screening programmes aimed at assessing falls risk. The machine learning analyses also revealed a high degree of shared information between certain domains and significant redundancy within the single-domain analyses. From a practical perspective, the results support the need for a multi-domain approach incorporating functional and physical measures to fully capture the complexity of falls in older women.

### Single-domain results

The findings of the single-domain analyses suggest that it is possible to distinguish between fallers and non-fallers with a high degree of accuracy using a multivariate approach for the balance, gait and strength measures (Table 3). Interestingly, with respect to predicting who was going to fall, the clinical and body composition domains did not perform as well as the other domains, and only demonstrated acceptable discriminatory ability between groups (with leave-one-out cross validation accuracies of 59% to 70%). Although some of the body composition and clinical measures variables were able to distinguish between fallers and non-fallers, other supplementary data may be required to achieve better discrimination. This finding is important for those settings which only have access to clinical measures or body composition variables when screening for falls risk. Although a total of 281 variables were included from the outset, the variable importance techniques identified significant redundancy within the data, with only 51 variables selected as important for the multi-domain analysis. This finding indicates that many of the included variables, which have been measured in previous studies, may be less informative when discriminating between groups. These results can, therefore, be used to optimise the design of falls screening protocols which are used to identify individuals at a high risk of falls and provide targeted falls prevention interventions.

#### Balance domain

It is noticeable that the balance variables identified as important were associated with the more challenging protocols (e.g. limits of stability and unilateral stance) with these tasks playing an important role in many activities of daily living (e.g. walking, turning and reaching). These findings corroborate those of others who have reported that fallers exhibited greater instability compared with non-fallers when standing on one limb [74] and on foam with eyes closed [9]. However, the importance of several limits of stability variables is in contrast to some studies [31, 33, 75]. Fallers seemed to have a greater reliance on somatosensory inputs compared with non-fallers; this is a promising finding because this variable is easy to calculate if sway velocity data is being collected on a firm and foam surface. Melzer et al. (2004) [31] suggested that balance control in narrow stance is a useful tool for discriminating between fallers and non-fallers. As such, it was surprising that no variables were selected as important from the narrow stance protocols. It is important to note that instability increased for both groups during the narrow stance trials highlighting that these trials were challenging for both groups and may be unsuitable for falls discrimination purposes in older women.

#### Gait domain

The analysis of the gait measures indicated that a combination of spatiotemporal, kinematic, GRF, and variability variables can distinguish between fallers and non-fallers with an outstanding degree of accuracy (AUC≥0.90). The gait variables identified as being good discriminators were taken from both the UGS and MGS trials which supports the use of these conditions for screening purposes [52]. Overall, the important variables suggest that fallers adopted a more cautious gait strategy compared with non-fallers, with fallers exhibiting shorter steps, greater knee flexion at toe-off, a longer braking phase duration, and a more pronounced double support strategy. These observations may be indicative of reduced dynamic balance ability [76] and weight acceptance ability [77] in fallers. For some of the gait measures, fallers demonstrated increased variability, suggesting that they walked with an inconsistent gait pattern [78]. On the other hand, fallers demonstrated lower variability for some variables, suggesting that a degree of variability is necessary for maintaining dynamic balance [79], although this may demonstrate the availability of fewer strategies to deal with gait instability and perturbations [80]. Several gait variables appeared redundant when discriminating between groups, such as mid-stance knee angle (an estimation of foot clearance during swing) and propulsive impulse. Considering these variables would seem important for the navigation of obstacles and control of speed during the gait cycle [81], it may be that both groups exhibited age-related declines (independent of falls history) which reduced the sensitivity of these variables for falls discrimination.

#### Strength domain

Several peak torque variables, namely knee flexion, dorsiflexion and plantar flexion, were identified as important discriminators which is in agreement with previous investigations that have reported lower maximal strength in fallers at the knee and ankle [34, 40]. Considering the key role of these muscles during activities of daily living [46], the reduced strength capacity of fallers likely contributed to the balance [82] and gait differences [83] observed between the two groups. Three asymmetry variables were identified as important discriminators, although an inconsistent pattern was found across different muscle groups and contraction types [84]. Whilst more research is needed to fully understand the patterns of strength asymmetry in elderly older women, the measurement of asymmetry should be considered in research and clinical practice. The variable importance analysis highlighted some variables that were not important in discriminating between the two groups. These included knee extension peak torque, knee extensor RTD and trunk strength. These findings likely reflect the contrasting research that exists regarding the discriminatory ability of RTD [40, 85] and trunk strength [86, 87] that may result from differences in the measurement protocols (i.e. muscle group, contraction type) that have been adopted. Nevertheless, the present findings highlight that maximal strength variables for the knee flexors, dorsiflexors and plantar flexors may be more important than maximal or rapid strength variables for the knee extensors or trunk muscles for inclusion in falls screening protocols.

#### Clinical measures

A combination of variables measured during the TUG, POMA and gait speed protocols appeared useful when differentiating between groups. Both the TUG and POMA incorporate a range of movements used during daily living, which may explain why these clinical measures were identified as important. Although previous studies have also reported significant differences between fallers and non-fallers for TUG time [88, 89], the optimal cut-off threshold (7.85 s) in this work was quicker than most of the previous literature and values used in clinical settings [90]. This suggests that quicker cut-off thresholds may be necessary to improve the classification accuracy of the TUG. A novel aspect of this study was the inclusion of gait speed reserve [52], with fallers demonstrating a greater capacity to increase walking speed relative to their UGS. Interestingly, performance during the chair stand and stair tests were not important discriminators despite their similarities with activities of daily living [91]. From this, it may be suggested that these measures are not chosen for discrimination purposes in this population.

#### Body composition domain

The results showed that MQ appears to be an important discriminator between fallers and non-fallers. The findings concur with the few studies that have analysed this variable and reported fallers as having poorer MQ compared with non-fallers [92]. MQ has previously been associated with gait and functional performance in older adults [60, 93], and in this way, lower MQ in fallers may have contributed to poorer performance in gait and clinical measures variables. Total fat mass was also selected as an important discriminator, with fallers demonstrating higher fat mass compared with non-fallers as observed previously [94]. Increased body fat may be associated with greater intramuscular fat infiltration which can impair muscle function leading to declines in muscle strength [95]; however, DXA scans are unable to detect fat infiltration in muscle [96]. Although the use of DXA is not without its limitations [29], it is routinely used in this population, and the findings demonstrate the discriminatory sensitivity of segmental MQ measures which are easily obtained from these scans. In terms of BMD, the femoral strength index was selected as an important discriminator, with fallers demonstrating lower values for the this compared with non-fallers, suggesting poorer bone strength and an increased risk of hip fracture from a fall on the greater trochanter [97]. Given that the femoral strength index is a composite variable which integrates BMD and structural parameters and is adjusted for body size [98], it may provide more insight than individual measures of bone structure and geometry alone, which were not identified as important.

### Multi-domain results

The multi-domain findings indicate that a combination of 18 variables from the gait, clinical measures, strength and body composition domains (Table 6) are the most important discriminators between fallers and non-fallers in a multi-domain context and can distinguish between groups with a high degree of accuracy (Table 7). Whilst the findings support the need for a multi-domain approach to fully capture the complexity of falls in older women [6], the single-domain models presented in the gait and balance data analyses slightly outperformed the models in the multi-domain analysis, which is an unexpected finding. As such, this suggests that clinicians who work in settings where it is possible to measure the important gait and/or balance variables have the potential to predict likely fallers and non-fallers with a high degree of accuracy.

In terms of domain importance, the results show that the gait domain appears to play a dominant role in discriminating between fallers and non-fallers. Although there are only a limited number of previous studies which have adopted a comprehensive multi-domain approach [17, 18], the available studies support our finding that gait variables provide valuable insight into falls risk. This could be because walking is a complex movement pattern, underpinned by the sensory, nervous and musculoskeletal systems, incorporating postural control (static and dynamic) and mobility [99]. In addition to the importance of the gait domain, TUG time and POMA total score were also selected from the clinical domain. This is a promising finding given the accessibility of these tests, making them suitable for use in many settings. These measures, alongside gait variables, may provide important information because they capture multiple aspects of balance and mobility that are key for daily living activities. In terms of physical characteristics analysed, several strength variables measured during the dorsiflexion and knee flexion trials were also selected as important discriminators. Alongside these strength measures, thigh MQ is another key discriminating variable which may underpin differences observed in the gait and clinical measures variables between fallers and non-fallers [60, 92]. Although previous multi-domain studies [17, 18] have not included a comprehensive range of muscle function measures, the present findings indicate that certain variables (i.e. maximal strength, strength asymmetry, MQ) should be considered in falls screening procedures.

The variable importance analyses also facilitated identification of less important variables which were not needed to discriminate between groups, despite being selected from the single-domain analyses. Indeed, only one body composition variable (thigh MQ [combined torque]) and no balance variables were selected as important for the refined multi-domain dataset despite their high degree of accuracy in the single-domain analyses. It is important to note that the balance variables were largely measured during static balance tasks that may not be reflective of situations which occur during fall events [100]. Whilst this may have impacted the discriminatory sensitivity of the balance variables within the multi-domain analyses, the use of PLSCA provided novel insights into the shared information between domains (Table 10). This PLSCA revealed a considerable amount of shared information between the variables within the balance, gait and clinical measures domains. This may explain why, once the gait and clinical measures variables were included in the analyses, the balance variables were no longer needed to discriminate between groups in the multi-domain context. The same can be said for the body composition domain which showed a high level of shared information with the strength domain. In this respect, the findings provide unique information that will assist in selecting and prioritising tests and variables for falls risk screening which can be adapted depending on the accessibility of different measurement tools. Finally, the study demonstrates that application of PLSCA has considerable potential as a tool for undertaking discriminatory analysis and variable importance within large, complex datasets such as those relating to falls risk in a specific population.

### Limitations

Although the general applicability of these results is perhaps limited to community-dwelling older women who were healthy and relatively active, it is known that women are at an increased risk of falls compared with men resulting in calls for gender-specific analyses [101]. Future studies should be conducted in other populations, for example recruiting older men or women who are older and frailer than the sample used in this work. Falls status in this study was defined retrospectively which increases the potential for recall bias. However, this is common practice in a research and community setting and falls history is known to be one of the best predictors of future falls [77]. Another limitation of this work was that medication usage was not considered in the exclusion criteria, which might have had an impact given that some medications can affect balance. The sample size of the study was relatively small and whilst this limited the range of techniques that could be used (excluding, for example, hold-out validation and cluster analysis), the inclusion of cross-validation within the random forest and LOOCV methods meant that this remains one of the most comprehensive studies in this area to date. Although this study included a large set of 281 variables from across five domains, it should be acknowledged that the use of alternative tests (e.g. dynamic posturography, hand-grip dynamometry), protocols (e.g. dual-task gait conditions) and measurement techniques (e.g. magnetic resonance imaging) may produce different results and could be explored in future studies. Finally, missing values within the multi-domain data set were imputed to allow the machine learning techniques to be used. Whilst there are limitations associated with data imputation [102], PPCA has been shown to be favourable over other data imputation methods [103].

## Conclusions

This study demonstrates that it is possible to discriminate between older female fallers and non-fallers using a refined combination of variables and a multivariate machine learning approach. The findings illustrate the ability to distinguish between groups with a high degree of accuracy using a combination of variables from the gait, clinical measures, strength and body composition domains. As such, this suggests that it should be possible to develop models in the future that can predict with great accuracy who is likely to fall using just a few carefully chosen variables. However, the results of the study suggest that a multi-domain approach incorporating functional and physical characteristics will be necessary to fully capture the complexity of falls in older women. Notably, it is apparent that some domains (gait and strength) appear to play a more dominant role in differentiating between fallers and non-fallers, whilst other domains (e.g. balance) appear less important. From a screening perspective, the important variables identified can be used to inform the design of appropriate testing protocols for use in community and clinical settings when screening for falls risk in older women. This information can also be used to inform targeted falls prevention interventions for this population as well as variable and test selection when monitoring intervention effectiveness. The machine learning analyses revealed a high degree of shared information between certain domains and significant redundancy within single-domain analyses. From a practical perspective, this suggests that data collection with older women in community, clinical and research settings could be made more efficient by focusing on variables which are more informative in discriminating between fallers and non-fallers.

## Supporting information

S1 Checklist STROBE statement—checklist of items that should be included in reports of observational studies. (DOCX)

S1 Appendix Machine learning methods. (DOCX)

S1 Table Descriptive statistics for the mCTSIB, narrow stance, unilateral stance and weight-bearing squat data in the balance data package. (DOCX)

S2 Table Descriptive statistics for the limits of stability data included in the balance data package. (DOCX)

S3 Table Descriptive statistics for the UGS variables included in the gait data package. (DOCX)

S4 Table Descriptive statistics for the MGS variables included in the gait data package. (DOCX)

S5 Table MAD percentages for UGS variables included in the gait data package. (DOCX)

S6 Table MAD percentages for MGS variables included in the gait data package. (DOCX)

S7 Table Descriptive statistics for the variables included in the clinical measures data package. (DOCX)

S8 Table Descriptive statistics for the variables included in the strength data package. (DOCX)

S9 Table Descriptive statistics for the variables included in the body composition data package. (DOCX)
