# Supplementary material for: A machine learning approach to identify important variables for distinguishing between fallers and non-fallers in older women
Source: PLoS One. 2023 Oct 31;18(10):e0293729. doi: 10.1371/journal.pone.0293729 (PMC10617741; doi:10.1371/journal.pone.0293729)
Supplement: S1 Checklist — (DOCX) [file pone.0293729.s001.docx]

STROBE Statement—checklist of items that should be included in reports of observational studies

|  | Item No. | Recommendation | Page  No. | Relevant text from manuscript |
| --- | --- | --- | --- | --- |
| **Title and abstract** | 1 | (*a*) Indicate the study’s design with a commonly used term in the title or the abstract | 2 | As such, this cross-sectional study aimed to identify those functional variables (i.e. balance, gait and clinical measures) and physical characteristics (i.e. strength and body composition) that could best distinguish between older female fallers and non-fallers, using a novel machine learning approach. |
|  |  | (*b*) Provide in the abstract an informative and balanced summary of what was done and what was found | 2 | Overall, 60 community-dwelling older women (≥65 years), retrospectively classified as fallers or non-fallers, attended three data collection sessions. Data (281 variables) collected from tests in five separate domains (balance, gait, clinical measures, strength and body composition) were analysed using random forest and leave-one-variable-out partial least squares correlation analysis to assess variable importance. The strongest discriminators from each domain were then assessed in a multi-domain analysis using the same strategy. The machine learning approach coupled with receiver operating characteristics analysis identified that it is possible to distinguish between fallers and non-fallers with a high degree of accuracy (area under curve >0.80) using a combination of 18 variables from four domains, with the gait and strength domains being particularly informative for screening programmes aimed at assessing falls risk. |
| Introduction | | | |  |
| Background/rationale | 2 | Explain the scientific background and rationale for the investigation being reported | 2-6 | See introduction section for full details. |
| Objectives | 3 | State specific objectives, including any prespecified hypotheses | 6 | As such, this study sought to: a) identify the functional and physical factors that best differentiate between fallers and non-fallers in older women; b) quantify the relative importance of these variables; and c) identify redundant variables to inform future testing and screening procedures. |
| Methods | | | |  |
| Study design | 4 | Present key elements of study design early in the paper | 8-9 | A cross-sectional study design was employed within this research. Participants attended three data collection sessions in the Carnegie Research Institute at Leeds Beckett University. Recruitment for this study opened in January 2017, and data collection took place between March 2017 and December 2018, with visits approximately two months apart. During session one, participants underwent screening of baseline characteristics, clinical measures, and balance assessment. During session two, gait analysis was performed, and during the final session, body composition and strength measurements were conducted (Fig 1). |
| Setting | 5 | Describe the setting, locations, and relevant dates, including periods of recruitment, exposure, follow-up, and data collection | 8-9 | Participants attended three data collection sessions in the Carnegie Research Institute at Leeds Beckett University. |
| Participants | 6 | (*a*) *Cohort study*—Give the eligibility criteria, and the sources and methods of selection of participants. Describe methods of follow-up  *Case-control study*—Give the eligibility criteria, and the sources and methods of case ascertainment and control selection. Give the rationale for the choice of cases and controls  *Cross-sectional study*—Give the eligibility criteria, and the sources and methods of selection of participants | 6-7 | Participants were randomly recruited from within the local community through a range of avenues, including liaising with Neighbourhood Network Schemes and the University of the Third Age. To advertise this study, several recruitment presentations were conducted and flyers were distributed physically and online (via email and social media). |
|  |  | (*b*) *Cohort study*—For matched studies, give matching criteria and number of exposed and unexposed  *Case-control study*—For matched studies, give matching criteria and the number of controls per case | N/A | N/A |
| Variables | 7 | Clearly define all outcomes, exposures, predictors, potential confounders, and effect modifiers. Give diagnostic criteria, if applicable | 13-18 | 281 variables were included in the analysis. See manuscript text for full details. |
| Data sources/ measurement | 8* | For each variable of interest, give sources of data and details of methods of assessment (measurement). Describe comparability of assessment methods if there is more than one group | 9-12 | 281 variables were included in the analysis. See manuscript text for full details. |
| Bias | 9 | Describe any efforts to address potential sources of bias | N/A | N/A |
| Study size | 10 | Explain how the study size was arrived at | 6 | The sample size was deemed appropriate given the exploratory nature of the work and aligns with recent research (e.g. [18]) that has conducted a comprehensive multi-domain analyses using machine learning techniques. |

Continued on next page

| Quantitative variables | 11 | Explain how quantitative variables were handled in the analyses. If applicable, describe which groupings were chosen and why | 13-18 | 281 variables were included in the analysis. See manuscript text for full details. |
| --- | --- | --- | --- | --- |
| Statistical methods | 12 | (*a*) Describe all statistical methods, including those used to control for confounding | 18-21 | See full details in manuscript text. |
|  |  | (*b*) Describe any methods used to examine subgroups and interactions | 18-21 | See full details in manuscript text. |
|  |  | (*c*) Explain how missing data were addressed | 21 | As such, any missing values were imputed using the Probabilistic Principal Component Analysis (PPCA) technique [59]. |
|  |  | (*d*) *Cohort study*—If applicable, explain how loss to follow-up was addressed  *Case-control study*—If applicable, explain how matching of cases and controls was addressed  *Cross-sectional study*—If applicable, describe analytical methods taking account of sampling strategy | N/A | N/A |
|  |  | (*e*) Describe any sensitivity analyses | N/A | N/A |
| Results | | | | |
| Participants | 13* | (a) Report numbers of individuals at each stage of study—eg numbers potentially eligible, examined for eligibility, confirmed eligible, included in the study, completing follow-up, and analysed | N/A | N/A |
|  |  | (b) Give reasons for non-participation at each stage | N/A | N/A |
|  |  | (c) Consider use of a flow diagram | N/A | N/A |
| Descriptive data | 14* | (a) Give characteristics of study participants (eg demographic, clinical, social) and information on exposures and potential confounders | 7-8 | Table 1 |
|  |  | (b) Indicate number of participants with missing data for each variable of interest |  |  |
|  |  | (c) *Cohort study*—Summarise follow-up time (eg, average and total amount) | N/A | N/A |
| Outcome data | 15* | *Cohort study*—Report numbers of outcome events or summary measures over time | N/A | N/A |
|  |  | *Case-control study—*Report numbers in each exposure category, or summary measures of exposure | N/A | N/A |
|  |  | *Cross-sectional study—*Report numbers of outcome events or summary measures | Supplementary material | The full data and univariate results for the single-domain analyses are presented in S1-S9 Tables. |
| Main results | 16 | (*a*) Give unadjusted estimates and, if applicable, confounder-adjusted estimates and their precision (eg, 95% confidence interval). Make clear which confounders were adjusted for and why they were included | 22-34 | See manuscript text for full results details. |
|  |  | (*b*) Report category boundaries when continuous variables were categorized | N/A | N/A |
|  |  | (*c*) If relevant, consider translating estimates of relative risk into absolute risk for a meaningful time period | N/A | N/A |

Continued on next page

| Other analyses | 17 | Report other analyses done—eg analyses of subgroups and interactions, and sensitivity analyses | 32 | Relationships between domains results. |
| --- | --- | --- | --- | --- |
| Discussion | | | | |
| Key results | 18 | Summarise key results with reference to study objectives | 34 | The aims of this study were to: a) identify the functional and physical factors that can best differentiate between fallers and non-fallers in older women; b) quantify the relative importance of these factors; and c) identify redundant factors to inform future testing and screening procedures. The findings demonstrate that it is possible to discriminate between fallers and non-fallers with a high degree of accuracy using a refined set of variables drawn from several domains. The machine learning analyses also revealed a high degree of shared information between certain domains and significant redundancy within the single-domain analyses. From a practical perspective, the results support the need for a multi-domain approach incorporating functional and physical characteristics to fully capture the complexity of falls in older women. The data also suggest that data collection with older women in community, clinical and research settings could be more efficient by focusing on variables which are more informative in predicting who is likely to fall and who is not. |
| Limitations | 19 | Discuss limitations of the study, taking into account sources of potential bias or imprecision. Discuss both direction and magnitude of any potential bias | 41-42 | Although the general applicability of these results is perhaps limited to community-dwelling older women who were healthy and relatively active, it is known that women are at an increased risk of falls compared with men resulting in calls for gender-specific analyses [92]. Falls status in this study was defined retrospectively which increases the potential for recall bias. However, this is common practice in a research and community setting and falls history is known to be one of the best predictors of future falls [69]. The sample size of the study was relatively small and whilst this limited the range of techniques that could be used (excluding, for example, hold-out validation and cluster analysis), the inclusion of cross-validation within the random forest and LOOCV methods meant that this remains one of the most comprehensive studies in this area to date. Although this study included a large set of 281 variables from across five domains, it should be acknowledged that the use of alternative tests (e.g. dynamic posturography, hand-grip dynamometry), protocols (e.g. dual-task gait conditions) and measurement techniques (e.g. magnetic resonance imaging) may produce different results and could be explored in future studies. Finally, missing values within the multi-domain data set were imputed to allow the machine learning techniques to be used. Whilst there are limitations associated with data imputation [93], PPCA has been shown to be favourable over other data imputation methods [94]. |
| Interpretation | 20 | Give a cautious overall interpretation of results considering objectives, limitations, multiplicity of analyses, results from similar studies, and other relevant evidence | 34-41 | See full details in discussion. |
| Generalisability | 21 | Discuss the generalisability (external validity) of the study results | 34-41 | See full details in discussion. |
| Other information | |  | | |
| Funding | 22 | Give the source of funding and the role of the funders for the present study and, if applicable, for the original study on which the present article is based | N/A | See funding section of manuscript. |

*Give information separately for cases and controls in case-control studies and, if applicable, for exposed and unexposed groups in cohort and cross-sectional studies.

**Note:** An Explanation and Elaboration article discusses each checklist item and gives methodological background and published examples of transparent reporting. The STROBE checklist is best used in conjunction with this article (freely available on the Web sites of PLoS Medicine at http://www.plosmedicine.org/, Annals of Internal Medicine at http://www.annals.org/, and Epidemiology at http://www.epidem.com/). Information on the STROBE Initiative is available at www.strobe-statement.org.
