# Supplementary material for: A machine learning approach to identify important variables for distinguishing between fallers and non-fallers in older women
Source: PLoS One. 2023 Oct 31;18(10):e0293729. doi: 10.1371/journal.pone.0293729 (PMC10617741; doi:10.1371/journal.pone.0293729)
Supplement: S1 Appendix — (DOCX) [file pone.0293729.s002.docx]

# S1 Appendix. Machine learning methods

# Random forests

Random forests are a robust ensemble classification technique, which are frequently used in machine learning [1]. Ensemble methods aggregate the results from a combination of models; this is beneficial as a single model could potentially overfit the data [2]. Random forests are resistant to overfitting (i.e. producing models that are applicable only to the particular dataset in question) and have none of the strict distributional assumptions required when using many traditional techniques [3].

A random forest is an ensemble of classification trees. Classification trees use recursive partitioning of a full dataset, splitting the data into smaller and more homogeneous subgroups, to fit classification models from many predictor variables [4]. The individual classification trees which are produced using bootstrap bagging, whereby bootstrap samples for each tree are randomly drawn from the original data, and each sample is used to train an individual classification tree [1]. The random forest models presented in this work are based on 500 trees [5]. The bootstrap samples contain the same number of observations as the original data and are drawn with replacement [4]. This means that an observation can be selected more than once in the bootstrap sample, whilst some observations will not be selected. Each observation has an equal probability of being selected during each random draw. As a result of the bootstrap bagging approach, ~63% of observations are used when training each tree, meaning that ~37% of observations are absent from any given sample [1]. These absent observations are commonly referred to as the out-of-bag (OOB) samples, and the OOB samples are used as test data for their corresponding tree to estimate the prediction error [4, 6].

In addition to the bootstrap sampling, another element of randomness is achieved using random variable selection. Rather than splitting a tree using the best split from all the predictor variables (which would result in a group of similar trees), the random forest algorithm selects a random subset of variables at each level of the tree, which are drawn without replacement, and then chooses the variable from this subset which produces the best split [4]. These random elements of the algorithm reduce the correlation between trees and the risk of overfitting the data. In this work, the number of predictor variables tested at each split was equal to the square root of the total number of variables in the model [7]. Of the random predictors chosen at each level of the tree, the variable resulting in the optimal split in the data is selected based on maximising purity between classes, a measure of how homogeneous the groups are. To decide this objectively, the variable with the lowest Gini index is chosen, which quantifies how well a potential split is separating the observations of the two classes at this level in the tree (i.e. the Gini index is a measure of impurity). Once the variable is chosen, this process is repeated for every stage of the tree until it reaches the largest possible size, and no further splits improve the Gini index. For each stage of the tree, a new random subset of predictor variables is chosen, these predictor variables can be used more than once throughout the levels of the tree. After completion of the tree, the whole process, starting with a new bootstrap sample, is repeated across 500 trees to build the forest.

## Random forest cross validation

The random forest algorithm, in the ‘randomForest’ library package in R, includes an internal validation technique. This technique can be completed using the OOB sample for each tree, meaning that a separate cross validation method is not needed [3]. As each tree is only fitted using approximately 63% of the data, the OOB data can be used to estimate the prediction error, referred to as the OOB error rate. To do this, the OOB data for a given tree are “dropped” down the tree, and each OOB observation is assigned the class of the final node at which the observation lands. This process is repeated across the forest, with each tree voting once for each of its OOB observations. Following this, the random forest class prediction for each observation was computed by taking the majority vote (aggregated across the forest) from trees for which the given observation was OOB. The OOB error rate is the misclassification error of the OOB samples across the forest [4]. The probability of the forest classifying each observation as a member of a certain class (i.e. faller or non-faller) was also calculated as the proportion of trees which predicted each class out of the total number of trees for which that observation was OOB [3].

## Random forest variable importance analysis

Random forests were also used to assess the relative importance of the predictor variables for differentiating between fallers and non-fallers, determined by the mean decrease in Gini index for each variable [8]. The decrease in Gini index for an individual variable split relates to the difference between the mean Gini index in the child nodes (weighted by the number of observations which reach each child node) and the Gini index of the parent node (the node one level above). Mean decrease in Gini index for a given variable is calculated as the average decrease in Gini index of every instance where that variable is used to split the data across the entire forest. The larger the mean decrease in Gini index, the more important the variable is discriminating between fallers and non-fallers.

Although mean decrease in Gini index is a robust measure, it is important to note that this can be biased in favour of variables with more distinct numerical values or fewer missing values. To overcome this, the Gini index was corrected for bias using a heuristic correction strategy [5]. For each random forest model, the mean decrease in Gini index for each variable in the model was plotted on a variable importance plot where the variables were ordered from most important (largest mean decrease in Gini index) to least important.

# Partial least squares correlation analysis

Partial least squares correlation analysis (PLSCA) was used to explore the relationships between the measured predictor variables and falls history [9]. PLSCA is a technique used in neuroimaging which analyses associations between two sets of data, and can handle large and complex datasets [10]. The tool underpinning PLSCA is singular value decomposition (SVD); therefore, immunising this technique from multicollinearity issues [11]. PLSCA can also be used with datasets that include more predictor variables than observations. PLSCA establishes the strength of any relationships between two subgroups of data using SVD to determine the inertia of the covariance matrix.

For each data package, a baseline PLSCA model containing all predictor variables was constructed. Initially, all data were mean centred and standardised to unit variance [10], before being partitioned into two subgroups (matrices): matrix X contained the indicator variable (falls history), and matrix Y contained the measured predictor variables. The relationships between the falls indicator and the predictor variables were then stored in a covariance matrix (matrix R), calculated using Equation 1.

Equation 1

$$R = Y^{T}X$$

SVD was then performed on matrix R, decomposing this matrix into three orthogonal matrices (denoted U, S and V). Matrix U contains the left singular vectors, matrix S is a diagonal matrix containing the singular values, and matrix V contains the right singular vectors. SVD was computed using Equation 2.

Equation 2

$$R=USV^{T}$$

Following SVD on matrix R, the amount of shared information between the falls indicator and the predictor variables was determined by calculating the singular value inertia using Equation 3 [10].

Equation 3

$$Inertia = \sum_{i}^{n} s_{i}$$

Where: n is the number of non-zero elements of matrix S, and s_i_ is the i^th^ diagonal element of matrix S.

The measured inertia is an effect size indicative of the strength of the relationship between the falls indicator and the predictor variables, with a greater value representing more shared information between these subgroups [10]. The latent variables of matrix Y (i.e. linear combinations of the original variables) were calculated by projecting the original variables onto their respective left singular vectors using Equation 4.

Equation 4

$${Latent}_{Y} = YU$$

The statistical significance of the measured inertia was computed using a permutation test, during which the rows of matrix Y were randomly permuted 10,000 times [10], replacing any relationship between the subgroups with a random configuration. For each permutation of matrix Y, the PLSCA process described above was repeated, producing 10,000 values for the singular value inertia. These inertia values were plotted against the frequency of their occurrence during the 10,000 repetitions, providing the distribution of inertia which could occur by chance (i.e. the null distribution). From this, the statistical significance of the measured inertia was determined by calculating the *p* value (i.e. the proportion of permutations which resulted in a level of inertia greater than or equal to the measured inertia).

## Leave-one-variable-out PLSCA

To quantify the relative importance of the predictor variables, the leave-one-variable-out (LOVO) technique was employed using the baseline PLSCA model, and the methods proposed by Weaving at al. (2019) [11]. This technique allowed in-depth interrogation of the data to establish which predictor variables were most influential, and therefore important when differentiating between fallers and non-fallers. The LOVO technique involved an iterative process whereby the PLSCA was repeated several times after construction of the baseline model which included all the predictor variables. On each repetition of the analysis, a different predictor variable was omitted from matrix Y, and the change in singular value inertia from the baseline model was quantified [11]. This allowed the evaluation of the relative contribution of each predictor variable to falls history, with a small change representing a less important variable and a larger change representing an influential variable, and therefore an important predictor of falls history.

After this process was repeated for all variables, a variable importance plot was produced showing the decrease in singular value inertia attributed to each predictor, with variables ordered from most important to least important.

# Logistic regression

Binomial multiple logistic regression analyses were performed to produce several models to distinguish between fallers and non-fallers. Since there were still more predictor variables than participants in most of the data packages, logistic regression could only be performed on the refined datasets, once the redundant variables had been omitted. To assess multicollinearity in each logistic regression model, the variance inflation factor (VIF) was calculated for each predictor [12]. The VIF shows how much the variance of the coefficient estimate is being inflated by multicollinearity, with values exceeding 10 being indicative of multicollinearity and values exceeding 4 a cause for concern [11, 12].

The baseline logistic regression models were refined to improve model fit and the best models were selected using several approaches. Initially, the model was refined by hand using a backwards stepwise procedure, excluding non-significant (*p*>0.10) predictor variables [4]. The baseline model was also refined using an automatic backwards step function, based on minimising the Akaike information criterion (AIC). Finally, the model was refined using an in-house algorithm which assessed all possible combinations of the predictor variables to identify the model with the minimum Bayesian information criterion (BIC). The AIC and BIC are statistical approaches which consider how well a given model fits the data alongside how complex the model is [7]. In some data packages, these model selection criteria resulted in the same refined models. The class prediction probabilities for each observation from the logistic regression were fed into receiver operator curve (ROC) analyses, so that area under the curve (AUC), sensitivity, specificity and optimum cut-off thresholds could be determined.

## Logistic regression leave-one-out cross validation

To determine the general applicability of each logistic regression model (i.e. to test how each model performed on unseen data), and to check that the models were not overfitting, leave-one-out cross validation (LOOCV) was performed [13]. Overfitting the data results in a model which performs poorly with unseen data and therefore, one which is not particularly useful [14]. Consequently, it is important to assess the general applicability of these models using cross validation methods [15]. Following pilot work, the LOOCV method was deemed most appropriate for the models presented in this thesis. This is supported by previous work which has suggested that LOOCV performs well with small sample sizes and produces comparable results to 10-fold cross validation methods [15].

The LOOCV procedure is an iterative process which involves splitting the observations into two subgroups, which are different for each iteration. The test set (one observation) and the training set (the remaining observations). Following this, the logistic regression model was fit on the training set, and a prediction was made for the observation in the test set to evaluate the model’s performance [14]. Once the prediction was made, the mean squared error was calculated as the square of the difference between the prediction and the observed value of the observation in the test set. This process was repeated for a number of times equal to the number of observations, with a different observation included in the test set on each iteration [15]. Finally, the mean squared error from each iteration was averaged to provide a LOOCV estimate for the model.

# References

1. Breiman L. Random forests. Machine learning. 2001;45(1): 5-32.

2. Liaw A, Wiener M. Classification and regression by randomForest. R news. 2002;2(3): 18-22.

3. Pecl GT, Tracey SR, Danyushevsky L, Wotherspoon S, Moltschaniwskyj NA. Elemental fingerprints of southern calamary (Sepioteuthis australis) reveal local recruitment sources and allow assessment of the importance of closed areas. Can J Fish Aquat Sci. 2011;68(8): 1351-1360.

4. Heidema AG, Boer J, Nagelkerke N, Mariman E, van der A DL, Feskens EJ. The challenge for genetic epidemiologists: how to analyze large numbers of SNPs in relation to complex diseases. BMC genetics. 2006;7(1): 1-15.

5. Carpita M, Sandri M, Simonetto A, Zuccolotto P. Football Mining with R. In: Zhao Y, Cen Y, editors. Data Mining Applications with R. Orlando: Academic Press; 2013. pp. 397–433.

6. Huang BF, Boutros PC. The parameter sensitivity of random forests. BMC bioinformatics. 2016;17(1): 1-13.

7. Hastie T, Tibshirani R, Friedman J. The elements of statistical learning: data mining, inference, and prediction. Springer series in statistics. Springer New York. 2009.

8. Calle ML, Urrea V. Letter to the editor: Stability of Random Forest importance measures. Brief Bioinform. 2011;12(1): 86-89.

9. McIntosh A, Bookstein F, Haxby JV, Grady C. Spatial pattern analysis of functional brain images using partial least squares. Neuroimage. 1996;3(3): 143-157.

10. Krishnan A, Williams LJ, McIntosh AR, Abdi H. Partial Least Squares (PLS) methods for neuroimaging: a tutorial and review. Neuroimage. 2011;56(2): 455-475.

11. Weaving D, Jones B, Ireton M, Whitehead S, Till K, Beggs CB. Overcoming the problem of multicollinearity in sports performance data: A novel application of partial least squares correlation analysis. PLoS ONE. 2019;14(2): e0211776.

12. Midi H, Sarkar SK, Rana S. Collinearity diagnostics of binary logistic regression model. Journal of interdisciplinary mathematics. 2010;13(3): 253-267.

13. Stone M. Cross‐validatory choice and assessment of statistical predictions. J R Stat Soc Series B Methodol. 1974;36(2): 111-133.

14. James G, Witten D, Hastie T, Tibshirani R. An introduction to statistical learning: with applications in R: Spinger; 2013.

15. Molinaro AM, Simon R, Pfeiffer RM. Prediction error estimation: a comparison of resampling methods. Bioinformatics. 2005;21(15): 3301-3307.
