# Supplementary material for: A machine learning approach to identify important variables for distinguishing between fallers and non-fallers in older women
Source: PLoS One. 2023 Oct 31;18(10):e0293729. doi: 10.1371/journal.pone.0293729 (PMC10617741; doi:10.1371/journal.pone.0293729)
Supplement: S1 Table — (DOCX) [file pone.0293729.s003.docx]

**S1 Table.** **Descriptive statistics for the mCTSIB, narrow stance, unilateral stance and weight-bearing squat data in the balance data package.**

|  | **Fallers (n=15)** | **Non-fallers (n=25)** | ***p* value** | **ES** |
| --- | --- | --- | --- | --- |
| **mCTSIB** | | | | |
| Firm EO SV (°/s) | 0.31±0.12 | 0.32±0.08 | 0.94 | 0.03 |
| Firm EC SV (°/s) | 0.41±0.15 | 0.43±0.15 | 0.62 | 0.16 |
| Foam EO SV (°/s) | 0.63±0.12 | 0.61±0.13 | 0.65 | 0.15 |
| Foam EC SV (°/s) | 1.60±0.45 | 1.43±0.32 | 0.21 | 0.46 |
| Firm RR | 1.43±0.56 | 1.39±0.40 | 0.81 | 0.09 |
| Foam RR | 2.62±0.76 | 2.44±0.76 | 0.48 | 0.24 |
| EO SR | 2.30±0.97 | 2.00±0.53 | 0.29 | 0.41 |
| EC SR | 4.23±1.45 | 3.55±1.08 | 0.13 | 0.55 |
| **Narrow stance** | | | | |
| NS firm EO SV (°/s) | 0.55±0.16 | 0.56±0.14 | 0.89 | 0.04 |
| NS firm EC SV (°/s) | 0.93±0.35 | 1.00±0.28 | 0.52 | 0.22 |
| NS foam EO SV (°/s) | 0.89±0.17 | 0.93±0.20 | 0.44 | 0.24 |
| NS foam EC SV (°/s) | 3.10±0.85 | 3.02±1.30 | 0.82 | 0.07 |
| NS firm RR | 1.69±0.54 | 1.84±0.56 | 0.42 | 0.27 |
| NS foam RR | 3.63±1.36 | 3.28±1.11 | 0.40 | 0.29 |
| NS EO SR | 1.71±0.55 | 1.73±0.45 | 0.91 | 0.04 |
| NS EC SR | 3.68±1.33 | 3.20±1.36 | 0.28 | 0.36 |
| **Unilateral stance** | | | | |
| Left EO SV (°/s) | 1.66±1.94 | 1.38±0.91 | 0.61 | 0.20 |
| Left EC SV (°/s) | 5.56±3.32 | 6.42±2.76 | 0.41 | 0.29 |
| Right EO SV (°/s) | 1.67±1.13 | 1.23±0.36 | 0.16 | 0.60 |
| Right EC SV (°/s) | 7.47±3.71 | 5.90±2.49 | 0.18 | 0.50 |
| Composite EO SV (°/s) | 1.67±1.48 | 1.30±0.53 | 0.37 | 0.37 |
| Composite EC SV (°/s) | 6.48±3.08 | 6.16±2.07 | 0.72 | 0.13 |
| Symmetry angle EO (%) | 10.16±7.60 | 7.49±8.98 | 0.32 | 0.31 |
| Symmetry angle EC (%) | 13.59±11.46 | 12.10±9.30 | 0.67 | 0.15 |
| **Weight-bearing squat** | | | | |
| Symmetry angle 0° (%) | 4.06±3.02 | 4.01±3.22 | 0.96 | 0.02 |
| Symmetry angle 30° (%) | 5.06±4.28 | 4.90±4.18 | 0.91 | 0.04 |
| Symmetry angle 60° (%) | 6.33±6.61 | 5.13±5.28 | 0.56 | 0.21 |

EC: eyes closed; EO: eyes open; ES, effect size; mCTSIB, Modified Clinical Test of Sensory Integration on Balance; NS, narrow stance; RR: Romberg ratio; SR: somatosensory ratio; SV: sway velocity.

Data are presented mean ± SD.

* *p≤*0.10, ** *p≤*0.05, *** *p≤*0.001.
