# Supplementary material for: A machine learning approach to identify important variables for distinguishing between fallers and non-fallers in older women
Source: PLoS One. 2023 Oct 31;18(10):e0293729. doi: 10.1371/journal.pone.0293729 (PMC10617741; doi:10.1371/journal.pone.0293729)
Supplement: S2 Table — (DOCX) [file pone.0293729.s004.docx]

**S2 Table. Descriptive statistics for the limits of stability data included in the balance data package.**

|  | **Fallers (n=15)** | **Non-fallers (n=25)** | ***p* value** | **ES** |
| --- | --- | --- | --- | --- |
| **Anterior** | | | | |
| Reaction time (s) | 0.73±0.25 | 0.70±0.31 | 0.70 | 0.12 |
| Movement velocity (°/s) | 4.21±1.76 | 4.96±2.49 | 0.28 | 0.33 |
| Endpoint excursion (%) | 66.33±19.44 | 73.44±15.68 | 0.24 | 0.41 |
| Maximum excursion (%) | 84.60±15.17 | 94.60±16.52 | 0.06* | 0.62 |
| Directional control (%) | 83.20±7.95 | 84.08±9.32 | 0.75 | 0.10 |
| **Right** | | | | |
| Reaction time (s) | 0.80±0.34 | 0.79±0.37 | 0.92 | 0.03 |
| Movement velocity (°/s) | 5.75±2.90 | 4.96±2.66 | 0.40 | 0.28 |
| Endpoint excursion (%) | 61.87±23.95 | 68.92±15.89 | 0.32 | 0.37 |
| Maximum excursion (%) | 81.07±15.96 | 84.68±10.50 | 0.44 | 0.28 |
| Directional control (%) | 80.60±8.98 | 73.68±11.12 | 0.04** | 0.67 |
| **Posterior** | | | | |
| Reaction time (s) | 0.75±0.28 | 0.73±0.36 | 0.83 | 0.06 |
| Movement velocity (°/s) | 2.93±1.69 | 2.91±1.58 | 0.96 | 0.02 |
| Endpoint excursion (%) | 48.00±13.48 | 39.40±9.25 | 0.04** | 0.78 |
| Maximum excursion (%) | 70.47±18.76 | 63.96±18.38 | 0.29 | 0.35 |
| Directional control (%) | 63.93±22.58 | 55.52±20.03 | 0.24 | 0.40 |
| **Left** | | | | |
| Reaction time (s) | 0.82±0.35 | 0.72±0.26 | 0.33 | 0.35 |
| Movement velocity (°/s) | 4.34±1.76 | 4.79±2.12 | 0.47 | 0.23 |
| Endpoint excursion (%) | 72.87±20.26 | 70.08±13.98 | 0.64 | 0.17 |
| Maximum excursion (%) | 85.60±13.43 | 85.44±10.37 | 0.97 | 0.01 |
| Directional control (%) | 81.00±8.87 | 77.80±11.75 | 0.34 | 0.30 |
| **Composite** | | | | |
| Reaction time (s) | 0.78±0.20 | 0.74±0.16 | 0.50 | 0.23 |
| Movement velocity (°/s) | 4.31±1.48 | 4.41±1.77 | 0.85 | 0.06 |
| Endpoint excursion (%) | 62.27±9.34 | 62.96±8.33 | 0.81 | 0.08 |
| Maximum excursion (%) | 80.43±9.18 | 82.17±8.63 | 0.56 | 0.20 |
| Directional control (%) | 77.18±7.83 | 72.77±8.76 | 0.11 | 0.52 |

ES, Effect Size.

Data are presented mean ± 1SD. Group differences and effect sizes were determined using two-tailed *t*-tests and Cohen’s *d* for continuous variables.

* *p≤*0.10, ** *p≤*0.05, *** *p≤*0.001.
