# Supplementary material for: A machine learning approach to identify important variables for distinguishing between fallers and non-fallers in older women
Source: PLoS One. 2023 Oct 31;18(10):e0293729. doi: 10.1371/journal.pone.0293729 (PMC10617741; doi:10.1371/journal.pone.0293729)
Supplement: S3 Table — (DOCX) [file pone.0293729.s005.docx]

**S3 Table.** **Descriptive statistics for the UGS variables included in the gait data package.**

|  | **Fallers (n=17)** | **Non-fallers (n=23)** | ***p* value** | **ES** |
| --- | --- | --- | --- | --- |
| Contact time (s) | 0.62±0.05 | 0.61±0.05 | 0.42 | 0.26 |
| Weight Acceptance Peak Force (BW) | 1.20±0.10 | 1.21±0.08 | 0.81 | 0.08 |
| Mid-stance Peak Force (BW) | 0.66±0.09 | 0.63±0.07 | 0.23 | 0.42 |
| Push-off Peak Force (BW) | 1.16±0.07 | 1.16±0.09 | 0.92 | 0.03 |
| Vertical Peak Force (BW) | 1.22±0.09 | 1.23±0.08 | 0.66 | 0.15 |
| Time to Weight Acceptance Peak Force (s) | 0.14±0.02 | 0.13±0.02 | 0.16 | 0.47 |
| Time to Mid-stance Peak Force (s) | 0.30±0.03 | 0.29±0.02 | 0.11 | 0.54 |
| Time to Push-off Peak Force (s) | 0.47±0.03 | 0.46±0.03 | 0.37 | 0.29 |
| Weight Acceptance Rate (BW/s) | 9.20±2.13 | 9.25±1.69 | 0.93 | 0.03 |
| Push-off Rate (BW/s) | -9.25±1.46 | -10.09±3.59 | 0.32 | 0.29 |
| Braking Peak Force (BW) | -0.23±0.05 | -0.25±0.03 | 0.15 | 0.50 |
| Propulsion Peak Force (BW) | 0.25±0.04 | 0.25±0.03 | 0.99 | 0.00 |
| Braking force impulse (BWs) | -0.03±0.01 | -0.03±0.00 | 0.79 | 0.09 |
| Propulsion force impulse (BWs) | 0.03±0.01 | 0.03±0.00 | 0.62 | 0.17 |
| Braking phase duration (s) | 0.32±0.03 | 0.29±0.03 | 0.01** | 0.88 |
| Propulsion phase duration (s) | 0.31±0.03 | 0.32±0.03 | 0.14 | 0.47 |
| Change in horizontal velocity (m/s) | 0.00±0.01 | 0.00±0.01 | 0.50 | 0.22 |
| Step length index | 0.43±0.03 | 0.47±0.03 | 0.001*** | 1.06 |
| Step frequency (Hz) | 2.06±0.13 | 2.07±0.13 | 0.72 | 0.11 |
| HS ankle angle (°) | 101±3 | 99±4 | 0.20 | 0.41 |
| HS knee angle (°) | 172±6 | 172±5 | 0.69 | 0.13 |
| TO ankle angle (°) | 121±6 | 122±6 | 0.46 | 0.24 |
| TO knee angle (°) | 127±4 | 131±5 | 0.01** | 0.87 |
| MS trunk angle (°) | 84±2 | 86±3 | 0.11 | 0.50 |
| MS knee angle (°) | 113±4 | 114±5 | 0.54 | 0.19 |

ES, effect size; HS, heel strike; MS, mid-stance; TO, toe-off.

Data are presented mean ± SD.

* *p≤*0.10, ** *p≤*0.05, *** *p≤*0.001.
