# Supplementary material for: A machine learning approach to identify important variables for distinguishing between fallers and non-fallers in older women
Source: PLoS One. 2023 Oct 31;18(10):e0293729. doi: 10.1371/journal.pone.0293729 (PMC10617741; doi:10.1371/journal.pone.0293729)
Supplement: S4 Table — (DOCX) [file pone.0293729.s006.docx]

**S4 Table.** **Descriptive statistics for the MGS variables included in the gait data package.**

|  | **Fallers (n=17)** | **Non-fallers (n=23)** | ***p* value** | **ES** |
| --- | --- | --- | --- | --- |
| Contact time (s) | 0.51±0.05 | 0.52±0.05 | 0.34 | 0.31 |
| Weight Acceptance Peak Force (BW) | 1.43±0.16 | 1.41±0.13 | 0.73 | 0.12 |
| Mid-stance Peak Force (BW) | 0.48±0.11 | 0.45±0.10 | 0.39 | 0.28 |
| Push-off Peak Force (BW) | 1.15±0.10 | 1.17±0.11 | 0.46 | 0.24 |
| Vertical Peak Force (BW) | 1.43±0.16 | 1.42±0.12 | 0.91 | 0.04 |
| Time to Weight Acceptance Peak Force (s) | 0.09±0.03 | 0.09±0.02 | 0.98 | 0.01 |
| Time to Mid-stance Peak Force (s) | 0.25±0.03 | 0.26±0.03 | 0.39 | 0.28 |
| Time to Push-off Peak Force (s) | 0.38±0.04 | 0.40±0.04 | 0.28 | 0.36 |
| Weight Acceptance Rate (BW/s) | 18.93±9.27 | 16.72±6.64 | 0.41 | 0.28 |
| Push-off Rate (BW/s) | -11.03±1.79 | -11.22±1.77 | 0.74 | 0.11 |
| Braking Peak Force (BW) | -0.30±0.05 | -0.33±0.05 | 0.11 | 0.51 |
| Propulsion Peak Force (BW) | 0.29±0.04 | 0.29±0.04 | 0.75 | 0.10 |
| Braking force impulse (BWs) | -0.03±0.01 | -0.03±0.01 | 0.22 | 0.40 |
| Propulsion force impulse (BWs) | 0.03±0.01 | 0.04±0.01 | 0.12 | 0.51 |
| Braking phase duration (s) | 0.23±0.04 | 0.23±0.03 | 0.71 | 0.13 |
| Propulsion phase duration (s) | 0.28±0.02 | 0.30±0.04 | 0.08* | 0.53 |
| Change in horizontal velocity (m/s) | 0.00±0.01 | 0.00±0.01 | 0.29 | 0.34 |
| Step length index | 0.49±0.03 | 0.52±0.04 | 0.01** | 0.91 |
| Step width (m) | 0.12±0.05 | 0.11±0.06 | 0.49 | 0.21 |
| Step frequency (Hz) | 2.41±0.21 | 2.32±0.20 | 0.22 | 0.40 |
| HS ankle angle (°) | 96±3 | 97±4 | 0.61 | 0.16 |
| HS knee angle (°) | 167±5 | 168±6 | 0.63 | 0.15 |
| TO ankle angle (°) | 123±5 | 124±6 | 0.45 | 0.24 |
| TO knee angle (°) | 132±4 | 135±5 | 0.04** | 0.65 |
| MS trunk angle (°) | 84±2 | 85±3 | 0.21 | 0.40 |
| MS knee angle (°) | 114±5 | 114±4 | 0.94 | 0.03 |

ES, effect size; HS, heel strike; MS, mid-stance; TO, toe-off.

Data are presented mean ± SD.

* *p≤*0.10, ** *p≤*0.05, *** *p≤*0.001.
