# Supplementary material for: A machine learning approach to identify important variables for distinguishing between fallers and non-fallers in older women
Source: PLoS One. 2023 Oct 31;18(10):e0293729. doi: 10.1371/journal.pone.0293729 (PMC10617741; doi:10.1371/journal.pone.0293729)
Supplement: S5 Table — (DOCX) [file pone.0293729.s007.docx]

**S5 Table.** **MAD percentages for UGS variables included in the gait data package.**

|  | **Fallers (n=17)** | **Non-fallers (n=23)** | ***p* value** | **ES** |
| --- | --- | --- | --- | --- |
| Contact time | 1.18±0.84 | 1.11±0.55 | 0.77 | 0.10 |
| F1 | 2.04±1.18 | 1.74±0.92 | 0.39 | 0.29 |
| F2 | 3.03±1.82 | 2.12±1.54 | 0.10* | 0.55 |
| F3 | 1.23±0.79 | 1.18±0.69 | 0.84 | 0.07 |
| PVF | 1.83±1.04 | 1.61±0.77 | 0.46 | 0.25 |
| TF1 | 4.44±2.11 | 4.19±5.58 | 0.84 | 0.06 |
| TF2 | 2.61±1.91 | 1.72±0.89 | 0.09* | 0.63 |
| TF3 | 1.14±0.95 | 1.06±0.54 | 0.74 | 0.12 |
| WAR | 6.82±5.85 | 5.36±5.74 | 0.44 | 0.25 |
| POR | 2.86±2.58 | 2.26±1.34 | 0.39 | 0.31 |
| F4 | 6.33±4.22 | 4.18±2.49 | 0.07* | 0.65 |
| F5 | 3.18±1.94 | 3.29±2.41 | 0.87 | 0.05 |
| Braking force impulse | 6.69±3.90 | 5.48±3.07 | 0.30 | 0.35 |
| Propulsion force impulse | 3.69±3.16 | 3.72±3.16 | 0.97 | 0.01 |
| Braking phase duration | 2.82±1.97 | 5.36±3.12 | 0.003** | 0.94 |
| Propulsion phase duration | 2.84±1.82 | 3.42±1.49 | 0.29 | 0.35 |
| Step length index | 1.74±1.51 | 1.28±0.49 | 0.24 | 0.44 |
| Step frequency | 0.89±0.52 | 0.78±0.54 | 0.56 | 0.19 |
| HS ankle angle | 0.72±0.77 | 0.68±0.40 | 0.86 | 0.06 |
| HS knee angle | 0.72±0.44 | 0.56±0.34 | 0.22 | 0.42 |
| TO ankle angle | 0.76±0.37 | 1.07±0.70 | 0.07* | 0.54 |
| TO knee angle | 0.99±0.42 | 0.86±0.58 | 0.44 | 0.24 |
| MS trunk angle | 0.84±0.54 | 0.65±0.50 | 0.27 | 0.36 |
| MS knee angle | 1.35±0.86 | 1.45±0.91 | 0.71 | 0.12 |

ES, effect size; F1, weight acceptance peak force; F2; mid-stance peak force; F3, push-off peak force; F4, braking peak force; F5, propulsion peak force; HS, heel strike; MAD, median absolute deviation; MS, mid-stance; POR, push-off rate; TF1, time to weight acceptance peak force; TF2, time to mid-stance peak force; TF3, time to push-off peak force; TO, toe-off; VPF, vertical peak force; WAR, weight acceptance rate. Data are presented mean ± 1SD. Group differences and effect sizes were determined using two-tailed *t*-tests and Cohen’s *d* for all variables.

* *p≤*0.10, ** *p≤*0.05, *** *p≤*0.001.
