# Supplementary material for: A machine learning approach to identify important variables for distinguishing between fallers and non-fallers in older women
Source: PLoS One. 2023 Oct 31;18(10):e0293729. doi: 10.1371/journal.pone.0293729 (PMC10617741; doi:10.1371/journal.pone.0293729)
Supplement: S6 Table — (DOCX) [file pone.0293729.s008.docx]

**S6 Table.** **MAD percentages for MGS variables included in the gait data package.**

|  | **Fallers (n=17)** | **Non-fallers (n=23)** | ***p* value** | **ES** |
| --- | --- | --- | --- | --- |
| Contact time | 1.56±0.92 | 1.42±1.08 | 0.66 | 0.14 |
| F1 | 2.52±1.75 | 1.99±1.10 | 0.28 | 0.38 |
| F2 | 4.06±4.14 | 4.32±2.54 | 0.82 | 0.08 |
| F3 | 2.33±1.90 | 1.66±1.00 | 0.20 | 0.46 |
| PVF | 2.52±1.75 | 1.88±1.02 | 0.19 | 0.47 |
| TF1 | 4.03±2.42 | 4.85±5.96 | 0.55 | 0.17 |
| TF2 | 2.04±1.67 | 1.45±0.75 | 0.19 | 0.48 |
| TF3 | 1.81±1.07 | 1.45±1.36 | 0.35 | 0.29 |
| WAR | 5.84±3.75 | 8.71±9.07 | 0.18 | 0.39 |
| POR | 2.85±2.06 | 2.73±1.74 | 0.84 | 0.07 |
| F4 | 4.56±2.36 | 4.98±3.10 | 0.63 | 0.15 |
| F5 | 3.54±1.99 | 4.01±2.63 | 0.52 | 0.20 |
| Braking force impulse | 5.58±3.78 | 5.83±2.65 | 0.81 | 0.08 |
| Propulsion force impulse | 5.27±3.11 | 4.48±2.21 | 0.38 | 0.3 |
| Braking phase duration | 4.95±5.37 | 4.86±4.44 | 0.95 | 0.02 |
| Propulsion phase duration | 4.21±3.25 | 3.53±2.95 | 0.50 | 0.22 |
| Step length index | 1.28±1.11 | 1.50±0.96 | 0.52 | 0.22 |
| Step frequency | 0.61±0.55 | 1.29±0.93 | 0.01** | 0.86 |
| HS ankle angle | 0.92±0.63 | 0.97±0.50 | 0.80 | 0.09 |
| HS knee angle | 0.73±0.30 | 0.69±0.47 | 0.72 | 0.11 |
| TO ankle angle | 0.78±0.58 | 0.73±0.38 | 0.79 | 0.09 |
| TO knee angle | 1.17±0.58 | 0.76±0.50 | 0.03** | 0.77 |
| MS trunk angle | 0.70±0.48 | 0.70±0.38 | 0.97 | 0.01 |
| MS knee angle | 0.97±0.43 | 1.05±0.57 | 0.60 | 0.16 |

ES, effect size; F1, weight acceptance peak force; F2; mid-stance peak force; F3, push-off peak force; F4, braking peak force; F5, propulsion peak force; HS, heel strike; MAD, median absolute deviation; MS, mid-stance; POR, push-off rate; TF1, time to weight acceptance peak force; TF2, time to mid-stance peak force; TF3, time to push-off peak force; TO, toe-off; VPF, vertical peak force; WAR, weight acceptance rate. Data are presented mean ± 1SD. Group differences and effect sizes were determined using two-tailed *t*-tests and Cohen’s *d* for all variables.

* *p≤*0.10, ** *p≤*0.05, *** *p≤*0.001.
