# Supplementary material for: A machine learning approach to identify important variables for distinguishing between fallers and non-fallers in older women
Source: PLoS One. 2023 Oct 31;18(10):e0293729. doi: 10.1371/journal.pone.0293729 (PMC10617741; doi:10.1371/journal.pone.0293729)
Supplement: S7 Table — (DOCX) [file pone.0293729.s009.docx]

**S7 Table. Descriptive statistics for the variables included in the clinical measures data package.**

|  | **Fallers (n=18)** | **Non-fallers (n=28)** | ***p* value** | **ES** |
| --- | --- | --- | --- | --- |
| TUG time (s) | 8.30±1.18 | 7.55±1.11 | 0.04** | 0.66 |
| Stair ascent time (s) | 7.10±1.39 | 6.54±1.35 | 0.19 | 0.41 |
| Stair descent time (s) | 6.28±1.39 | 6.20±1.25 | 0.84 | 0.06 |
| CST performance (count) | 13±4 | 15±4 | 0.13 | 0.46 |
| UGS (m/s) | 1.43±0.13 | 1.52±0.14 | 0.04** | 0.62 |
| MGS (m/s) | 1.91±0.20 | 1.93±0.18 | 0.75 | 0.10 |
| Gait speed reserve | 1.34±0.14 | 1.27±0.08 | 0.08* | 0.62 |
| POMA balance score | 15.00±1.68 | 15.71±0.53 | 0.10* | 0.63 |
| POMA gait score | 11.33±0.91 | 11.61±1.03 | 0.35 | 0.28 |
| POMA total score | 26.33±1.75 | 27.32±1.25 | 0.05** | 0.68 |

CST, chair stand test; ES, effect size; MGS: maximal gait speed; POMA: Performance Oriented Mobility Assessment; TUG, Timed Up and Go; UGS, usual gait speed.

Data are presented mean ± SD.

* *p≤*0.10, ** *p≤*0.05, *** *p≤*0.001.
