# Supplementary material for: A machine learning approach to identify important variables for distinguishing between fallers and non-fallers in older women
Source: PLoS One. 2023 Oct 31;18(10):e0293729. doi: 10.1371/journal.pone.0293729 (PMC10617741; doi:10.1371/journal.pone.0293729)
Supplement: S8 Table — (DOCX) [file pone.0293729.s010.docx]

**S8 Table.** **Descriptive statistics for the variables included in the strength data package.**

|  | **Fallers (n=12)** | **Non-fallers (n=29)** | ***p* value** | **ES** |
| --- | --- | --- | --- | --- |
| **Isokinetic Trunk Strength** | | | | |
| *Trunk flexion 20°/s* |  | | | |
| PT (Nm/kg) | 1.11±0.39 | 1.13±0.29 | 0.87 | 0.06 |
| AngPT (°) | 54.17±15.76 | 51.34±13.90 | 0.60 | 0.20 |
| *Trunk flexion 45°/s* |  | | | |
| PT (Nm/kg) | 0.99 ±0.31 | 1.13±0.24 | 0.20 | 0.51 |
| AngPT (°) | 57.17±14.72 | 51.07±10.15 | 0.21 | 0.52 |
| *Trunk extension 20°/s* |  | | | |
| PT (Nm/kg) | 1.77±0.48 | 1.90±0.62 | 0.45 | 0.24 |
| AngPT (°) | 46.17±11.13 | 46.86±10.23 | 0.85 | 0.07 |
| *Trunk extension 45°/s* |  | | | |
| PT (Nm/kg) | 1.51±0.45 | 1.76±0.62 | 0.15 | 0.44 |
| AngPT (°) | 43.17±14.56 | 48.62±12.48 | 0.27 | 0.42 |
| *Flexion/extension ratios* |  | | | |
| 20°/s | 0.63±0.13 | 0.64±0.21 | 0.85 | 0.05 |
| 45°/s | 0.68±0.15 | 0.71±0.27 | 0.68 | 0.11 |
| **Isokinetic Knee Strength** | | | | |
| *Knee flexion 60°/s* |  | | | |
| DL PT (Nm/kg) | 0.75±0.14 | 0.83±0.12 | 0.07* | 0.69 |
| NDL PT (Nm/kg) | 0.65±0.15 | 0.73±0.11 | 0.10* | 0.68 |
| DL AngPT (°) | 51.5±11.68 | 48.38±13.16 | 0.46 | 0.24 |
| NDL AngPT (°) | 52.67±14.44 | 50.17±14.74 | 0.62 | 0.17 |
| SA (%) | 4.61±4.10 | 4.09±3.23 | 0.70 | 0.15 |
| *Knee flexion 120°/s* |  | | | |
| DL PT (Nm/kg) | 0.60±0.17 | 0.69±0.12 | 0.12 | 0.65 |
| NDL PT (Nm/kg) | 0.50±0.18 | 0.61±0.12 | 0.06* | 0.83 |
| DL AngPT (°) | 52.08±13.51 | 43.21±17.25 | 0.09* | 0.55 |
| NDL AngPT (°) | 52.17±13.35 | 54.28±16.53 | 0.67 | 0.13 |
| SA (%) | 7.03±5.16 | 4.01±2.68 | 0.08* | 0.85 |
| *Knee extension 60°/s* |  | | | |
| DL PT (Nm/kg) | 1.35±0.33 | 1.52±0.33 | 0.15 | 0.52 |
| NDL PT (Nm/kg) | 1.21±0.32 | 1.32±0.32 | 0.34 | 0.34 |
| DL AngPT (°) | 66.42±8.16 | 67.93±8.08 | 0.59 | 0.19 |
| NDL AngPT (°) | 67.25±7.40 | 68.59±6.88 | 0.60 | 0.19 |
| SA (%) | 3.65±4.87 | 4.68±4.00 | 0.52 | 0.24 |
| *Knee extension 120°/s* |  | | | |
| DL PT (Nm/kg) | 1.05±0.29 | 1.17±0.25 | 0.21 | 0.47 |
| NDL PT (Nm/kg) | 0.88±0.32 | 1.00±0.26 | 0.25 | 0.44 |
| DL AngPT (°) | 64.75±6.17 | 68.34±9.74 | 0.17 | 0.40 |
| NDL AngPT (°) | 69.25±10.84 | 65.52±9.68 | 0.31 | 0.37 |
| SA (%) | 6.10±4.63 | 5.39±4.51 | 0.66 | 0.16 |
| *H/Q ratios* |  | | | |
| 60°/s Left | 0.52±0.09 | 0.57±0.16 | 0.21 | 0.36 |
| 120°/s Left | 0.55±0.12 | 0.61±0.15 | 0.19 | 0.42 |
| 60°/s Right | 0.59±0.11 | 0.58±0.13 | 0.74 | 0.11 |
| 120°/s Right | 0.60±0.13 | 0.63±0.13 | 0.48 | 0.25 |
| **Isokinetic Ankle Strength** | | | | |
| *Dorsiflexion 60°/s* |  | | | |
| DL PT (Nm/kg) | 0.27±0.05 | 0.31±0.06 | 0.07* | 0.58 |
| NDL PT (Nm/kg) | 0.24±0.04 | 0.27±0.06 | 0.02** | 0.66 |
| DL AngPT (°) | 24.08±7.98 | 24.03±9.73 | 0.99 | 0.01 |
| NDL AngPT (°) | 25.92±9.33 | 27.07±8.30 | 0.71 | 0.13 |
| SA (%) | 4.26±3.65 | 3.98±3.37 | 0.82 | 0.08 |
| *Dorsiflexion 120°/s* |  | | | |
| DL PT (Nm/kg) | 0.19±0.05 | 0.24±0.05 | 0.02** | 0.82 |
| NDL PT (Nm/kg) | 0.18±0.04 | 0.21±0.06 | 0.05** | 0.62 |
| DL AngPT (°) | 23.92±10.15 | 28.62±8.26 | 0.17 | 0.53 |
| NDL AngPT (°) | 24.75±11.54 | 27.76±10.50 | 0.45 | 0.28 |
| SA (%) | 3.06±2.14 | 4.24±3.49 | 0.19 | 0.38 |
| *Plantar flexion 60°/s* |  | | | |
| DL PT (Nm/kg) | 0.66±0.24 | 0.76±0.22 | 0.24 | 0.44 |
| NDL PT (Nm/kg) | 0.54±0.19 | 0.62±0.21 | 0.19 | 0.44 |
| DL AngPT (°) | 7.08±7.72 | 6.28±8.31 | 0.77 | 0.10 |
| NDL AngPT (°) | 11.58±7.54 | 8.97±8.50 | 0.34 | 0.32 |
| SA (%) | 6.01±7.57 | 6.57±4.84 | 0.82 | 0.10 |
| *Plantar flexion 120°/s* |  | | | |
| DL PT (Nm/kg) | 0.43±0.19 | 0.53±0.14 | 0.12 | 0.65 |
| NDL PT (Nm/kg) | 0.35±0.15 | 0.42±0.12 | 0.16 | 0.56 |
| DL AngPT (°) | 11.25±8.37 | 6.93±8.02 | 0.14 | 0.53 |
| NDL AngPT (°) | 15.58±11.16 | 10.20±9.60 | 0.16 | 0.53 |
| SA (%) | 6.05±6.15 | 7.47±3.80 | 0.47 | 0.31 |
| *DF/PF ratios* |  | | | |
| 60°/s Left | 0.45±0.16 | 0.46±0.13 | 0.85 | 0.07 |
| 120°/s Left | 0.48±0.14 | 0.52±0.17 | 0.88 | 0.05 |
| 60°/s Right | 0.51±0.15 | 0.45±0.14 | 0.54 | 0.21 |
| 120°/s Right | 0.55±0.22 | 0.49±0.19 | 0.45 | 0.28 |
| **Isometric Knee Strength** | | | | |
| DL PT (Nm/kg) | 1.85±0.46 | 2.08±0.52 | 0.17 | 0.46 |
| NDL PT (Nm/kg) | 1.57±0.43 | 1.78±0.42 | 0.16 | 0.50 |
| PT SA (%) | 5.51±3.65 | 4.89±3.17 | 0.61 | 0.19 |
| Left RTD 0-50ms (Nm/s/kg) | 5.49±4.24 | 4.76±3.20 | 0.60 | 0.21 |
| Left RTD 0-100ms (Nm/s/kg) | 5.78±3.46 | 5.47±2.71 | 0.78 | 0.11 |
| Left RTD 0-200ms (Nm/s/kg) | 4.71±2.39 | 4.86±1.97 | 0.85 | 0.07 |
| Right RTD 0-50ms (Nm/s/kg) | 4.96±3.15 | 6.41±4.87 | 0.27 | 0.33 |
| Right RTD 0-100ms (Nm/s/kg) | 5.41±3.10 | 6.46±3.95 | 0.37 | 0.28 |
| Right RTD 0-200ms (Nm/s/kg) | 4.34±2.30 | 5.31±2.42 | 0.24 | 0.41 |
| RTD 0-50ms SA (%) | 13.56±7.89 | 16.44±9.37 | 0.32 | 0.32 |
| RTD 0-100ms SA (%) | 9.07±9.89 | 12.91±8.62 | 0.26 | 0.43 |
| RTD 0-200ms SA (%) | 12.15±10.65 | 10.44±6.92 | 0.61 | 0.21 |

AngPT, angle of peak torque; DF/PF, dorsiflexion / plantar flexion; DL, dominant limb; ES, effect size; H/Q, hamstrings/quadriceps; NDL, non-dominant limb; PT, peak torque; RTD, rate of torque development; SA, symmetry angle.

Data are presented mean ± 1SD.

* *p≤*0.10, ** *p≤*0.05, *** *p≤*0.001.
