# Supplementary material for: A machine learning approach to identify important variables for distinguishing between fallers and non-fallers in older women
Source: PLoS One. 2023 Oct 31;18(10):e0293729. doi: 10.1371/journal.pone.0293729 (PMC10617741; doi:10.1371/journal.pone.0293729)
Supplement: S9 Table — (DOCX) [file pone.0293729.s011.docx]

**S9 Table.** **Descriptive statistics for the variables included in the body composition data package.**

|  | **Fallers**  **(n=12)** | **Non-fallers (n=28)** | ***p* value** | **ES** |
| --- | --- | --- | --- | --- |
| **Fat mass** | | | | |
| Total FM (kg) | 25.00±8.60 | 20.53±6.54 | 0.13 | 0.62 |
| Percentage FM (%) | 38.51±6.41 | 35.04±6.89 | 0.14 | 0.51 |
| FMI (kg/m^2^) | 9.40±3.28 | 7.95±2.27 | 0.18 | 0.56 |
| FM SA (%) | 0.88±0.81 | 0.78±0.58 | 0.70 | 0.15 |
| VAT (kg) | 0.61±0.41 | 0.51±0.38 | 0.48 | 0.26 |
| **Lean tissue mass** | | | | |
| Total LM (kg) | 38.49±3.16 | 36.79±3.74 | 0.15 | 0.47 |
| Percentage LM (%) | 0.60±0.06 | 0.63±0.07 | 0.13 | 0.52 |
| LMI (kg/m^2^) | 14.51±1.61 | 14.34±1.13 | 0.75 | 0.13 |
| **Appendicular lean mass** | | | | |
| Total ALM (kg) | 16.65±1.79 | 15.71±2.12 | 0.16 | 0.47 |
| Percentage ALM (%) | 25.64±2.34 | 26.68±2.46 | 0.22 | 0.43 |
| ALMI (kg/m^2^) | 6.28±0.87 | 6.11±0.60 | 0.55 | 0.25 |
| ALMI SA (%) | 0.88±0.54 | 1.10±0.93 | 0.37 | 0.25 |
| Upper Body ALM (kg) | 3.80±0.39 | 3.76±0.60 | 0.79 | 0.08 |
| Lower Body ALM (kg) | 12.85±1.49 | 11.95±1.61 | 0.10* | 0.57 |
| **Bone mineral density** | | | | |
| Femoral neck BMD (g/cm^2^) | 0.82±0.09 | 0.79±0.11 | 0.39 | 0.27 |
| Upper neck BMD (g/cm^2^) | 0.63±0.08 | 0.62±0.10 | 0.82 | 0.07 |
| Lower neck BMD (g/cm^2^) | 1.01±0.10 | 0.96±0.13 | 0.21 | 0.40 |
| Ward’s triangle BMD (g/cm^2^) | 0.62±0.12 | 0.59±0.10 | 0.45 | 0.28 |
| Trochanter BMD (g/cm^2^) | 0.70±0.11 | 0.66±0.10 | 0.27 | 0.41 |
| Shaft BMD (g/cm^2^) | 1.01±0.13 | 0.98±0.14 | 0.51 | 0.23 |
| Total hip BMD (g/cm^2^) | 0.85±0.10 | 0.82±0.10 | 0.32 | 0.34 |
| **Hip structure** | | | | |
| Femoral neck T-score (SD) | -1.36±0.72 | -1.59±0.95 | 0.40 | 0.27 |
| Total hip T-score (SD) | -1.25±0.81 | -1.52±0.87 | 0.35 | 0.32 |
| HAL (mm) | 110.45±5.22 | 107.87±6.00 | 0.18 | 0.45 |
| FSI | 1.28±0.28 | 1.47±0.24 | 0.06* | 0.74 |
| Buckling ratio | 8.55±3.11 | 9.25±7.55 | 0.68 | 0.11 |
| Section modulus (cm^3^) | 502.32±77.87 | 511.33±95.34 | 0.76 | 0.10 |
| CSMI (mm^4^) | 9113.17±1825.91 | 9013.63±1952.15 | 0.88 | 0.05 |
| CSA (mm^2^) | 127.25±14.28 | 122.70±17.84 | 0.40 | 0.27 |
| **Thigh MQ (isometric KE torque)** | | | | |
| DL MQ (Nm/kg) | 30.58±5.79 | 33.49±7.30 | 0.19 | 0.42 |
| NDL MQ (Nm/kg) | 25.56±5.24 | 29.06±6.87 | 0.09* | 0.54 |
| Overall MQ (Nm/kg) | 28.07±5.28 | 31.28±6.92 | 0.12 | 0.49 |
| SA MQ (%) | 5.87±4.01 | 4.65±2.85 | 0.35 | 0.38 |
| **Thigh MQ (KE torque)** | | | | |
| DL MQ (Nm/kg) | 21.99±3.45 | 23.81±3.89 | 0.15 | 0.48 |
| NDL MQ (Nm/kg) | 19.93±4.06 | 21.28±4.31 | 0.35 | 0.32 |
| Overall MQ (Nm/kg) | 20.96±3.55 | 22.53±3.89 | 0.23 | 0.41 |
| SA MQ (%) | 3.51±4.58 | 3.90±4.14 | 0.80 | 0.09 |
| **Thigh MQ (combined torque)** | | | | |
| DL MQ (Nm/kg) | 34.27±4.17 | 36.69±4.98 | 0.13 | 0.51 |
| NDL MQ (Nm/kg) | 30.81±5.23 | 33.48±5.37 | 0.16 | 0.50 |
| Overall MQ (Nm/kg) | 32.54±4.44 | 35.08±4.95 | 0.12 | 0.53 |
| SA MQ (%) | 3.58±3.90 | 3.13±2.88 | 0.72 | 0.14 |
| **Shank MQ (PF torque)** | | | | |
| DL MQ (Nm/kg) | 25.16±7.55 | 29.71±8.11 | 0.10* | 0.57 |
| NDL MQ (Nm/kg) | 21.15±7.48 | 24.21±7.63 | 0.25 | 0.40 |
| Overall MQ (Nm/kg) | 23.21±6.94 | 26.96±7.64 | 0.14 | 0.50 |
| SA MQ (%) | 5.98±6.29 | 7.02±4.92 | 0.62 | 0.19 |
| **Shank MQ (combined torque)** | | | | |
| DL MQ (Nm/kg) | 35.97±8.74 | 40.67±9.59 | 0.14 | 0.50 |
| NDL MQ (Nm/kg) | 30.84±7.35 | 35.01±8.65 | 0.13 | 0.50 |
| Overall MQ (Nm/kg) | 33.41±7.67 | 37.89±8.83 | 0.12 | 0.53 |
| SA MQ (%) | 5.02±4.04 | 5.26±3.34 | 0.86 | 0.07 |

ALM, appendicular lean mass; ALMI, appendicular lean mass index; BMD, bone mineral density; CSA, cross-sectional area; CSMI, cross-sectional moment of inertia; DL, dominant limb; ES, effect size; FM, fat mass; FMI, fat mass index; FSI, Femoral Strength Index; HAL, hip axis length; KE, knee extension; LM, lean tissue mass; LMI, lean mass index; MQ, muscle quality; NDL, non-dominant limb; PF, plantar flexion; SA, symmetry angle; VAT, visceral adipose tissue.

Data are presented mean ± SD.

* *p≤*0.10, ** *p≤*0.05, *** *p≤*0.001.
